# The SWELL1 Channel Promotes Ischemic Brain Damage by Mediating Neuronal Swelling and Glutamate Toxicity

**DOI:** 10.1002/advs.202401085

**Published:** 2024-07-26

**Authors:** Jianan Chen, Junhua Yang, Jiachen Chu, Kevin Hong Chen, Jesse Alt, Rana Rais, Zhaozhu Qiu

**Affiliations:** ^1^ Department of Physiology Johns Hopkins University School of Medicine Baltimore MD 21205 USA; ^2^ Nanozyme Medical Center School of Basic Medical Sciences Zhengzhou University Zhengzhou 450001 China; ^3^ Department of Veterinary Integrative Biosciences School of Veterinary Medicine and Biomedical Sciences Texas A&M University College Station Texas TX 77843 USA; ^4^ Johns Hopkins Drug Discovery Johns Hopkins University School of Medicine Baltimore MD 21205 USA; ^5^ Solomon H. Snyder Department of Neuroscience Department of Neurosurgery Johns Hopkins University School of Medicine Baltimore MD 21205 USA

**Keywords:** cytotoxic neuronal swelling, dicumarol, glutamate excitotoxicity, ischemic stroke, SWELL1 channels

## Abstract

Cytotoxic neuronal swelling and glutamate excitotoxicity are two hallmarks of ischemic stroke. However, the underlying molecular mechanisms are not well understood. Here, it is reported that SWELL1, the essential subunit of the volume‐regulated anion channel (VRAC), plays a dual role in ischemic injury by promoting neuronal swelling and glutamate excitotoxicity. SWELL1 expression is upregulated in neurons and astrocytes after experimental stroke in mice. The neuronal SWELL1 channel is activated by intracellular hypertonicity, leading to Cl^−^ influx‐dependent cytotoxic neuronal swelling and subsequent cell death. Additionally, the SWELL1 channel in astrocytes mediates pathological glutamate release, indicated by increases in neuronal slow inward current frequency and tonic NMDAR current. Pharmacologically, targeting VRAC with a new inhibitor, an FDA‐approved drug Dicumarol, attenuated cytotoxic neuronal swelling and cell death, reduced astrocytic glutamate release, and provided significant neuroprotection in mice when administered either before or after ischemia. Therefore, these findings uncover the pleiotropic effects of the SWELL1 channel in neurons and astrocytes in the pathogenesis of ischemic stroke and provide proof of concept for therapeutically targeting it in this disease.

## Graphical abstract

This study reveals the critical involvement of the SWELL1 channel in ischemic brain injury pathogenesis, highlighting its role in cytotoxic neuronal swelling and astrocytic glutamate excitotoxicity. Notably, pharmacological inhibition of SWELL1 by the FDA‐approved drug Dicumarol effectively blocks these deleterious pathways, underscoring SWELL1 as a promising therapeutic target for ischemic stroke.

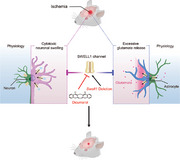

## 1 Introduction

Ischemic stroke is among the severest forms of acute brain injury, affecting millions of people annually worldwide.^[^ ^1^ ^]^ The primary brain injury after ischemia occurs as a result of an abrupt cessation of blood supply to an area of the brain, which triggers a cascade of deleterious events including glutamate excitotoxicity, cytotoxic neuronal swelling, mitochondrial disturbance, and oxidative stress, all of which promote secondary brain injury.^[^ ^2^, ^3^ ^]^ Currently, the mainstay of ischemic stroke therapy is rapid reperfusion with intravenous thrombolysis and endovascular thrombectomy, which reduce disability but are time‐critical.^[^ ^4^, ^5^ ^]^ Further understanding of the important mechanisms of stroke injury is required to provide new targets aimed at preventing deleterious pathways and curtailing secondary brain damage.^[^ ^6^, ^7^ ^]^

Multiple depolarizing triggers during stroke pathophysiology, including NMDA receptor activation and intense neuronal spiking, cause intracellular Na^+^ accumulation. This results in cytotoxic neuronal swelling and neuronal cell death, which can progress to brain edema, a pathological hallmark of ischemic stroke.^[^ ^8^ ^]^ Intracellular Na^+^ accumulation through activation of SUR1‐TRPM4 channels and glutamate receptors, or inhibition of Na^+^/K^+^‐ATPase, has been shown to be an early driver of cytotoxic neuronal edema.^[^ ^9^ ^]^ However, increasing intracellular Na^+^ concentration alone is not sufficient to cause persistent neuronal swelling. Subsequent Cl^−^ influx is a crucial step for the ultimate neuronal swelling and death. Although Cl^−^ transporters, SLC26A11 and Na^+^‐K^+^‐Cl^−^ co‐transporter 1 (NKCC1), have been implicated in mediating neuronal swelling,^[^ ^10^, ^11^ ^]^ the influx pathways for Cl^−^ remain poorly understood.^[^ ^9^ ^]^ Pharmacological studies have suggested the volume‐regulated anion channel (VRAC) as a potential candidate for Cl^−^ loading leading to cytotoxic neuronal swelling.^[^ ^12^ ^]^ However, because its molecular identity was unknown until recently, available evidence supporting a role for VRAC in this process was limited by the use of non‐specific small molecule inhibitors, which are known to affect the activity of many other ion channels and transporters.^[^ ^13^, ^14^, ^15^, ^16^ ^]^

We and others have identified SWELL1 (LRRC8A, a member of the leucine‐rich repeat‐containing family 8 proteins) as the only essential pore‐forming subunit of VRAC.^[^ ^17^, ^18^, ^19^ ^]^ SWELL1 forms hetero‐hexameric VRAC channels with its other four homologs (LRRC8B–E), the composition of which determines the channel's biophysical properties. These findings enable molecular studies to directly elucidate the role of this channel in Cl^−^ loading‐dependent cytotoxic neuronal swelling. SWELL1 is also expressed in astrocytes and can transport larger signaling molecules, including the principal excitatory neurotransmitter glutamate.^[^ ^19^ ^]^ In ischemic stroke, glutamate is largely released from astrocytes, which over‐activates N‐methyl‐D‐aspartate receptors (NMDARs) in neurons and causes excitotoxic neuronal death.^[^ ^20^ ^]^ We previously showed that the SWELL1 channel in astrocytes permeates glutamate in vitro and contributes to acute ischemic brain damage in mice.^[^ ^21^ ^]^ However, the underlying mechanism and its contribution to glutamate excitotoxicity during ischemic stroke have not been examined. More importantly, Nestin‐cre mediated brain‐wide deletion of SWELL1 has been shown to generate greater neuroprotection compared to deletion in astrocytes alone in experimental stroke,^[^ ^22^, ^23^ ^]^ although it also leads to lethality before 8 weeks of age.^[^ ^24^ ^]^ These genetic studies suggest that SWELL1 channels in astrocytes and neurons are both involved in ischemic brain damage.

In this study, using genetic and pharmacological approaches, we show that the SWELL1 channel contributes to ischemic stroke by simultaneously promoting two main injury pathways: cytotoxic neuronal swelling and glutamate excitotoxicity. Targeting the SWELL1 channel with Dicumarol, a novel blocker and FDA‐approved drug, alleviates both cellular injury pathways and provides neuroprotection after ischemic stroke. Our work establishes the SWELL1 channel as a key player in the injury cascade following ischemic stroke and as a potential novel pharmacological target for pleiotropic therapeutic intervention.

## 2 Results

### 2.1 Expression of SWELL1 in Neurons and Astrocytes is Upregulated After Acute Brain Ischemia

**Figure 1:**
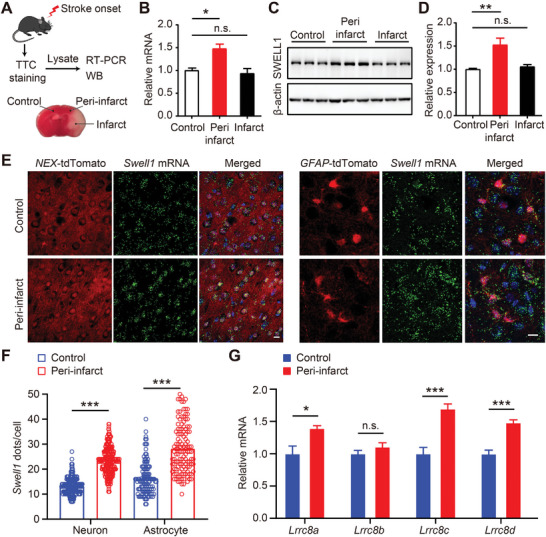
SWELL1 expression is upregulated in neurons and astrocytes of the peri‐infarct region. A) Schematic workflow for examining SWELL1 expression. B) RT‐PCR analysis of *Swell1* mRNA expression in regions of control (uninvolved hemisphere), peri‐infarct, and infarct core obtained 8 h after tMCAO. *n* = 3 mice for each group. Student's *t*‐tests, ^*^ *p* < 0.05. C) Western blot and D) quantification of SWELL1 protein expression 8 h after tMCAO. *n* = 3 mice for each group. Student's *t*‐tests, ^**^ *p *< 0.01. E) Representative images of *Swell1* RNAscope in situ hybridization (green) in corresponding cortical regions of the uninvolved hemisphere and peri‐infarct obtained 8 h after tMCAO. Neurons or astrocytes were labeled with tdTomato in *NEX*‐Ai14 or *GFAP*‐Ai14 mice, respectively. Scale bar, 10 µm. F) Quantification of *Swell1* expression in NEX‐ or GFAP‐ positive cells. *n* = 120–286 cells from 3 mice for each group. Student's *t*‐tests, ^***^ *p *< 0.001. G) RT‐PCR analysis of *Lrrc8b‐d* mRNA expression in regions of the control (uninvolved hemisphere) and peri‐infarct obtained 8 h after tMCAO. *n* = 3 mice for each group. student's *t*‐tests, ^*^ *p* < 0.05, ^***^ *p *< 0.001. Data are reported as mean ± SEM.

The expression of many ion channels and transporters increases after ischemia, further contributing to the pathogenesis of stroke.^[^ ^25^, ^26^ ^]^ To determine whether SWELL1 expression is regulated by ischemia, we first used quantitative real‐time PCR (RT‐PCR) and immunoblotting to analyze the expression of SWELL1 in three regions after transient middle cerebral artery occlusion (tMCAO): the uninvolved hemisphere, peri‐infarct region, and the infarct core, identified by 2,3,5‐triphenyltetrazolium chloride (TTC) staining (**Figure** **1**A). Consistent with previous reports,^[^ ^23^, ^27^ ^]^ the mRNA and protein levels of SWELL1 increased significantly after tMCAO in the TTC^+^ peri‐infarct region compared to the contralateral hemisphere and the TTC^−^ infarct core (Figure 1B–D), indicating that SWELL1 expression is upregulated in response to ischemia. As neurons and surrounding glial cells, including astrocytes and microglia, are critical to the pathogenesis of stroke,^[^ ^28^ ^]^ we sought to further determine which cell type upregulates *Swell1* after stroke. We labeled neurons or astrocytes by crossing Ai14 reporter with *NEX*‐cre (a mouse line expressing Cre‐recombinase in pyramidal neurons of the neocortex and hippocampus^[^ ^29^ ^]^) or *mGFAP*‐cre (a mouse line expressing Cre‐recombinase in astrocytes of the postnatal brain^[^ ^30^ ^]^) mice and performed RNAscope in situ hybridization in brain sections after tMCAO. Interestingly, both NEX^+^ neurons and GFAP^+^ astrocytes in the peri‐infarct region exhibited elevated *Swell1* expression compared to the undamaged side (Figure 1E,F), suggesting that neuronal and astrocytic SWELL1 channels may play an important role in the pathogenesis of ischemic stroke. *Swell1* expression level was comparable in Iba1^+^ microglial cells of the peri‐infarct and undamaged regions (Figure S1A, Supporting Information). This is consistent with a recent report that microglial SWELL1 is not involved in ischemic brain injury.^[^ ^31^ ^]^ In addition to SWELL1, other VRAC subunits (LRRC8B‐D) have also been shown to be highly expressed in the brain, except LRRC8E (Figure S1B, Supporting Information). Thus, we analyzed the expression changes of *Lrrc8b‐d* and found that the mRNA levels of *Lrrc8c* and *8d* were also significantly increased in the peri‐infarct region compared to the uninvolved hemisphere (Figure 1G). This suggests that the VRAC channel is upregulated in the peri‐infarct region and may play a role in ischemic brain injury.

### 2.2 SWELL1 Channel Contributes to Cytotoxic Neuronal Swelling and Cell Death

Na^+^ influx is known to serve as the driving force for Cl^−^ loading, leading to cytotoxic neuronal swelling in ischemic stroke.^[^ ^9^ ^]^ To test if the SWELL1 channel can be activated by intracellular hypertonicity with increased NaCl concentration, we first performed patch‐clamp recordings in HeLa cells with the normal internal solution plus additional NaCl. We observed the typical outwardly rectifying VRAC currents in WT cells, the amplitudes of which were correlated with the increasing NaCl concentrations (Figure S2A,B, Supporting Information). The currents were abolished in *SWELL1* knockout (KO) cells (Figure S2B,C, Supporting Information), indicating that intracellular Na^+^ increase activates SWELL1‐dependent VRAC currents.

**Figure 2:**
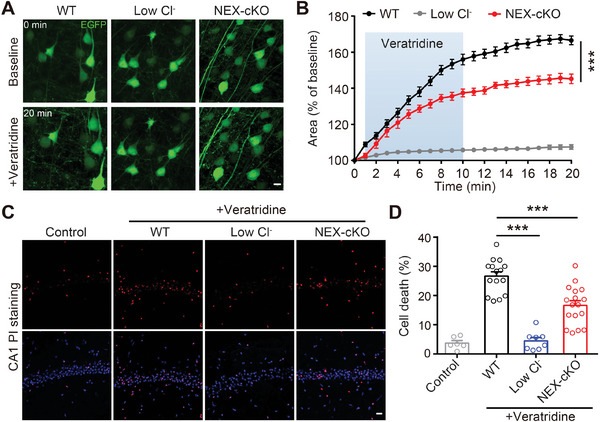
The SWELL1 channel contributes to cytotoxic neuronal swelling and cell death. A) Representative images of cortical neurons infected with pAAV‐CaMKIIα‐EGFP before and after treatment with 50 µm veratridine. Scale bar, 10 µm. B) Quantification of cross‐sectional area as a proxy for neuronal volume. *n* = 11–17 brain slices from 3–5 mice for each group. Two‐way ANOVA, Bonferroni post hoc test, ^***^ *p* < 0.001. C) Representative images of CA1 neurons stained with Hoechst 33342 (blue) for nuclei of all neurons and with propidium iodide (red) for nuclei of dead neurons. Scale bar, 10 µm. D) Percentage of veratridine‐induced neuronal cell death. *n* = 6–17 brain slices from 3–5 mice for each group. One‐way ANOVA, ^***^ *p* < 0.001. Data are reported as mean ± SEM.

To explore the role of the SWELL1 channel in cytotoxic neuronal swelling, we sparsely labeled cortical neurons in mouse brains with adeno‐associated viruses (AAVs) carrying CaMKIIα‐EGFP and performed live‐cell imaging in acute brain slices to measure the cross‐section area of labeled neurons, often used as a proxy for cell volume.^[^ ^10^ ^]^ Neuronal swelling was induced by bath application of veratridine (50 µm), which prolongs Na^+^ entry through voltage‐gated sodium channels by preventing their inactivation. Consistent with a previous report,^[^ ^10^ ^]^ application of veratridine triggered immediate and marked neuronal swelling even in the presence of a cocktail of blockers (20 µm DNQX, 100 µm D‐AP5, 30 µm Cadmium, and 100 µm picrotoxin) inhibiting Na^+^, Ca^2+^, and Cl^−^ entry pathways through glutamate‐gated AMPARs and NMDARs, and GABA‐activated Cl^−^ channels (**Figure** **2**A,B). Removing extracellular Cl^−^ dramatically inhibited the swelling of neurons, confirming that Cl^−^ entry is indeed required for neuronal swelling (Figure 2A,B). To examine the role of the SWELL1 channel in this process, we generated neuron‐specific *Swell1* KO mice using the *NEX*‐cre line (NEX‐cKO). We then prepared brain slices from NEX‐cKO mice for live‐cell imaging and confirmed the specific deletion of *Swell1* in CaMKIIα positive cortical neurons by RNAscope in situ hybridization (Figure S3, Supporting Information). Interestingly, genetic deletion of neuronal *Swell1* significantly reduced veratridine‐induced neuronal swelling (Figure 2A,B), indicating that the SWELL1 channel constitutes an important Cl^−^ loading pathway contributing to neuronal swelling. The residual neuronal swelling is likely due to the involvement of SLC26A11 and NKCC1.

Na^+^‐induced Cl^−^ influx and subsequent swelling can lead to cell death.^[^ ^10^, ^12^ ^]^ To test the role of the SWELL1 channel in inducing neuronal cell death, we applied veratridine in acute brain slices and performed propidium iodide (PI) staining to examine cell viability in the pyramidal cell layer of the hippocampal CA1 region, where the majority of cells are NEX‐positive. As expected, the application of veratridine for 10 min induced significant neuronal cell death after 1 h, even in the presence of a cocktail of blockers of glutamate‐gated AMPARs and NMDARs, and GABA‐activated Cl^−^ channels, whereas extracellular Cl^−^ removal abolished veratridine‐induced neuronal death (Figure 2C,D). These data indicate the necessity of Cl^−^ entry for both cytotoxic neuronal swelling and subsequent neuronal cell death. To investigate whether disruption of the SWELL1 channel reduces neuronal death, we prepared acute brain slices from NEX‐cKO mice and treated them with veratridine. Specific *Swell1* deletion in hippocampal CA1 neurons was confirmed by RNAscope in situ hybridization (Figure S4, Supporting Information). Consistent with the neuronal swelling results, we observed fewer PI‐positive nuclei in the CA1 pyramidal cell layer from NEX‐cKO mice compared to wild‐type (WT) littermate control mice (Figure 2C,D). Together, these data suggest that the SWELL1 channel plays a critical role in Cl^−^ influx‐dependent cytotoxic neuronal swelling and cell death.

### 2.3 Astrocytic Glutamate Release in the Peri‐Infarct Sites is Increased in a SWELL1 Channel‐Dependent Manner

**Figure 3:**
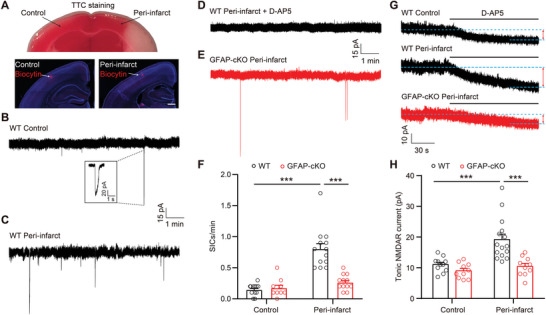
Astrocytic *Swell1* deletion leads to reduced SIC frequency and tonic NMDAR currents in the peri‐infarct region. A) Representative images of recorded cortical neurons in the control (uninvolved hemisphere) and the peri‐infarct region at 8 h after tMCAO. Scale bar, 500 µm. B,C) Representative traces of SIC recording from cortical neurons in control B) or the peri‐infarct region C) in brain slices from WT mice. Inset is one example of SIC. D,E) Representative traces of SIC recording from cortical neurons in the peri‐infarct region in brain slices from WT mice with bath perfusion of NMDAR antagonist D‐AP5 (50 µm) D) or from GFAP‐cKO mice E). F) Summary histogram of SIC frequency for WT (control: *n* = 12 cells from 4 mice; peri‐infarct: *n* = 13 cells from 4 mice) and GFAP‐cKO (control: *n* = 9 cells from 4 mice; peri‐infarct: *n* = 12 cells from 4 mice) mice. Two‐way ANOVA, Bonferroni post hoc test, ^***^ *p* < 0.001. G) Representative traces of tonic NMDAR currents recording from cortical neurons in the control and peri‐infarct region in brain slices from WT and GFAP‐cKO mice. Double arrows indicate the changes in baseline induced by application of the D‐AP5 (50 µm). H) Summary histogram of tonic NMDAR currents for WT (control: *n* = 11 cells from 4 mice; peri‐infarct: *n* = 15 cells from 4 mice) and GFAP‐cKO (control: *n* = 10 cells from 4 mice; peri‐infarct: *n* = 10 cells from 4 mice) mice. Two‐way ANOVA, Bonferroni post hoc test, ^***^ *p* < 0.001. Data are reported as mean ± SEM.

The SWELL1 channel in astrocytes has been proposed to be involved in ischemic stroke by releasing glutamate.^[^ ^32^ ^]^ We previously generated astrocyte‐specific *Swell1* knockout (GFAP‐cKO) mice and found that *Swell1* deletion in astrocytes significantly reduces brain infarcts and improves neurological outcomes after experimental stroke.^[^ ^21^ ^]^ To determine its underlying neuroprotective mechanism and the extent to which astrocytes employ the SWELL1 channel to release glutamate in ischemic stroke, we performed tMCAO surgery in WT mice and prepared acute brain slices after ischemia. We chose cortical neurons from the corresponding regions of the uninvolved hemisphere and peri‐infarct to record two types of extra‐synaptic NMDAR‐dependent currents with different characteristics (**Figure** **3**A). First, we measured the NMDAR‐dependent slow inward currents (SICs), as an indicator for astrocytic glutamate release.^[^ ^33^ ^]^ As expected, we observed a low SIC frequency in neurons from the uninvolved hemisphere (Figure 3B). However, the frequency was significantly increased in neurons from the peri‐infarct areas (Figure 3C,F). The SIC events were blocked by the NMDAR antagonist D‐AP5, indicating that they were stimulated by excessive glutamate after ischemic insult (Figure 3D). Second, we recorded the tonic NMDAR currents, which are activated by ambient extracellular glutamate released from astrocytes and modulate neuronal excitability due to their sustained nature.^[^ ^34^ ^]^ Similar to the SIC events, the tonic NMDAR currents, revealed by D‐AP5 application, were also significantly increased in the peri‐infarct areas compared to the uninvolved hemisphere (Figure 3 G,H). These data indicate that ischemic stroke induces a higher extracellular glutamate concentration in the peri‐infarct region. To determine whether SWELL1 channel‐mediated glutamate release contributes to the increased glutamate levels after stroke, we then performed tMCAO surgery on GFAP‐cKO mice and prepared acute brain slices for SIC and tonic NMDAR current recordings. As shown in Figure 3E‐H, both the SIC events and tonic NMDAR currents in the peri‐infarct region from GFAP‐cKO mice were significantly reduced compared to those in WT mice. These results suggest that the SWELL1 channel in astrocytes plays a major role in glutamate excitotoxicity.

### 2.4 Dicumarol, a Novel SWELL1 Channel Blocker, Inhibits Both Cytotoxic Neuronal Swelling and Glutamate Excitotoxicity

**Figure 4:**
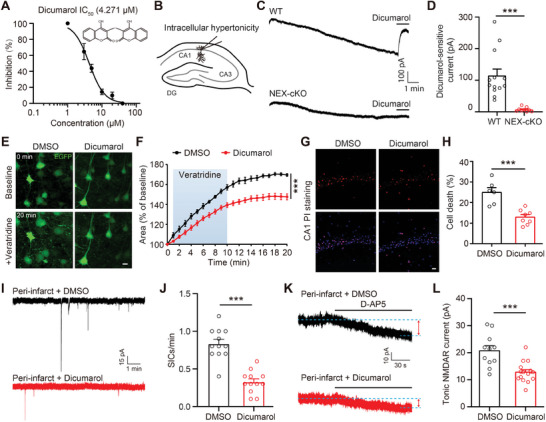
Dicumarol, a novel SWELL1 channel blocker, inhibits both cytotoxic neuronal swelling and glutamate excitotoxicity. A) Dose‐response curve of Dicumarol on SWELL1 currents, IC_50_ = 4.271 µm, *n* = 3–5 HeLa cells for each data point. B) Schematic illustration of whole cell‐patch technique in CA1 pyramidal neurons with normal internal solution plus 40 mm NaCl. (C) Representative current traces recorded at a holding potential of −70 mV from control neurons (top), and *Swell1* KO neurons (below) in murine brain slices. Dicumarol (40 µm) was added as indicated. D) Quantification of Dicumarol‐sensitive currents in control neurons (*n* = 13 cells from 3 mice), and *Swell1* KO neurons (*n* = 9 cells from 3 mice). Student's *t*‐tests, ^***^ *p* < 0.001. E) Representative images of cortical neurons infected with pAAV‐CaMKIIα‐EGFP before and after treatment with 50 µm veratridine. Scale bar, 10 µm. F) Quantification of neuronal cross‐sectional areas. *n* = 8–17 brain slices from 3–5 mice for each group. Two‐way ANOVA, Bonferroni post hoc test, ^***^ *p *< 0.001. G) Representative images of CA1 neurons stained with Hoechst 33342 (blue) for nuclei of all neurons and with propidium iodide (red) for nuclei of dead neurons. Scale bar, 10 µm. H) Percentage of veratridine‐induced neuronal cell death. *n* = 6–8 brain slices from 3 mice for each group. Student's *t*‐tests, ^***^ *p *< 0.001. I,K) Representative traces of SIC I) or tonic NMDAR current K) recorded from cortical neurons in the peri‐infarct areas in brain slices from WT mice 8 h after tMCAO with bath perfusion of DMSO (top) or 40 µM Dicumarol (below). Double arrows indicate the changes in baseline induced by application of D‐AP5 (50 µm). J,L) Summary histogram of SIC frequency in (J) or tonic NMDAR current in (L). *n* = 12–16 cells from 3 mice for each group. Student's *t*‐tests, ^***^ *p *< 0.001. Data are reported as mean ± SEM.

In addition to genetic approaches, we next aimed to investigate the mechanisms underlying the involvement of the SWELL1 channel in ischemic stroke using pharmacological tools. Repurposing existing drugs represents an efficient strategy to obtain potent compounds with fast translational potential. Toward this goal, we conducted a high‐throughput screen using the YFP quenching assay developed previously to report VRAC activity,^[^ ^17^ ^]^ and screened the NIH drug library (≈3000 FDA‐approved drugs). This screen led to the discovery of a new SWELL1 channel inhibitor, Dicumarol.^[^ ^35^ ^]^ Dicumarol inhibited, in a dose‐dependent manner, the hypotonicity‐activated SWELL1 currents in HeLa cells (**Figure** **4**A). Its half‐maximal inhibitory concentration (IC_50_) was ≈4 µm (Figure 4A), similar to the widely used SWELL1 channel inhibitor DCPIB (IC_50_: 5 µm),^[^ ^36^ ^]^ which is known to exhibit numerous off‐target effects.^[^ ^37^, ^38^, ^39^, ^40^ ^]^ To assess whether Dicumarol also inhibits SWELL1 channel‐mediated amino acid release, we assayed hypotonicity‐induced efflux of [^3^H]‐labeled taurine, an abundant intracellular amino acid derivative known to be mediated by the SWELL1 channel.^[^ ^17^ ^]^ Consistently, we observed a sharp increase in the rate of taurine efflux after hypotonic solution perfusion, which was dramatically blunted by Dicumarol treatment (Figure S5A, Supporting Information). These data suggest that Dicumarol not only blocks SWELL1 channel‐mediated Cl^−^ currents but also inhibits the release of larger organic osmolytes.

Next, we test whether the SWELL1 channel blocker Dicumarol can prevent cytotoxic neuronal swelling and glutamate excitotoxicity in ischemic stroke. We first examined whether Dicumarol can inhibit SWELL1 currents that are activated by intracellular hypertonicity. We performed patch‐clamp recordings using normal internal solution plus additional NaCl in WT HeLa cells. Similar to the genetic deletion of *SWELL1*, pharmacological inhibition of the channel by Dicumarol also abolished SWELL1 currents (Figure S5B,C, Supporting Information). To test if this inhibitory effect also happens in neurons, we performed whole‐cell recordings of hippocampal CA1 pyramidal neurons in brain slices from WT mice with the normal internal solution plus an additional 40 mM NaCl (Figure 4B). Consistent with the results in HeLa cells, we observed slowly developing inward SWELL1 currents, which were blocked by Dicumarol (Figure 4C). Under the same recording conditions, neurons in brain slices from NEX‐cKO mice did not develop obvious inward currents, and Dicumarol treatment showed no effect on the baseline currents (Figure 4C,D). These data suggest that intracellular Na^+^ accumulation activates Dicumarol‐sensitive SWELL1 currents in neurons. We further investigated the effect of Dicumarol on cytotoxic neuronal swelling by performing live‐cell imaging in acute brain slices from WT mice. Dicumarol treatment significantly reduced the magnitude of veratridine‐induced cell swelling in EGFP‐labeled neurons compared to DMSO controls (Figure 4E,F). Consistently, Dicumarol also inhibited veratridine‐induced neuronal cell death (Figure 4G,H). Taken together, these results indicate that Dicumarol attenuates cytotoxic neuronal swelling and cell death.

We then tested whether Dicumarol inhibits SWELL1 channel‐mediated glutamate release from astrocytes. To this end, we performed the sniffer‐patch experiment, as a sensitive functional bioassay for glutamate release from single astrocytes.^[^ ^21^, ^41^ ^]^ As illustrated in Figure S5D (Supporting Information), primary astrocytes isolated from WT mice were cultured as donor cells, and HEK293T cells were used as sensor cells after being transfected with a non‐desensitizing a‐amino‐3‐hydroxy‐5‐methyl‐4‐isoxazolepropionic acid (AMPA) receptor mutant, GluR1‐L497Y, which has a high glutamate affinity. The sensor cell was placed in direct contact with the donor astrocyte to detect glutamate efflux. By performing double whole‐cell patch‐clamp recordings, we observed that intracellular hypertonic solution (containing 5 mm glutamate) activated SWELL1 channels in the donor astrocytes, indicated by developing inward currents at −60 mV. Concurrently, inward AMPA receptor currents were recorded in the sensor cells, confirming astrocytic glutamate release (Figure S5E, Supporting Information). Using KO cells, we previously showed that this glutamate release is dependent on SWELL1 expression in the donor astrocytes.^[^ ^21^ ^]^ Acute treatment with Dicumarol inhibited SWELL1 currents in astrocytes (Figure S5E,F, Supporting Information). Importantly, the AMPA receptor currents in the sensor cells were also abolished (Figure S5E,G, Supporting Information). These results suggest that Dicumarol inhibits SWELL1 channel‐dependent glutamate efflux from astrocytes. To further examine the inhibitory effect of Dicumarol on SWELL1 channel‐mediated pathological glutamate release, we prepared acute brain slices from WT mice after tMCAO and measured SIC and tonic NMDAR currents with Dicumarol treatment. Compared to DMSO, Dicumarol treatment significantly reduced the ischemia‐induced SIC events and tonic NMDAR currents in the peri‐infarct region (Figure 4I–L), suggesting that the SWELL1 channel inhibitor Dicumarol blocks pathological glutamate release in an ex vivo setting.

Collectively, our results indicate that acute pharmacological inhibition of the SWELL1 channel effectively reduces cytotoxic neuronal swelling and glutamate excitotoxicity, thus phenocopying the effects of genetic deletion of *Swell1* in mice.

### 2.5 Dicumarol Provides Neuroprotection in Experimental Stroke Mouse Models

**Figure 5:**
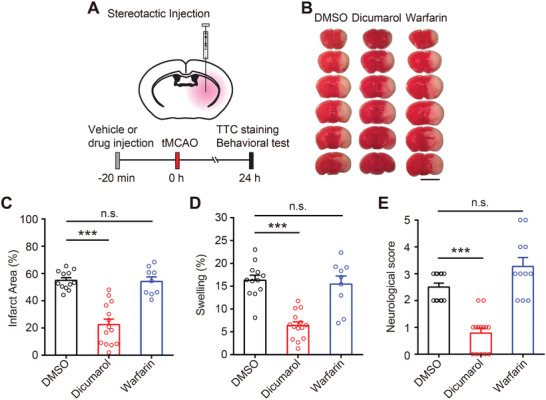
Dicumarol treatment before ischemia reduces brain damage in experimental stroke. A) Experimental design of the ischemic stroke study. 10 µL DMSO (1%), Dicumarol (100 µm in 1% DMSO), or Warfarin (100 µm in 1% DMSO) was infused into the brain 20 min before tMCAO surgery in male mice. Neurological deficit scores were evaluated 1 day after tMCAO, and brain samples were then collected for TTC staining. B) Representative images of TTC staining 1 day after tMCAO. Scale bar, 5 mm. C,D) Quantification of total infarct area volume C) and hemispheric swelling D) in (B). *n* = 9–14 mice for each group. One‐way ANOVA, ^***^ *p *< 0.001. E) Quantification of neurological deficit scores 1 day after tMCAO. *n* = 11–14 mice for each group. Kruskal–Wallis test, ^***^ *p *< 0.001. Data are reported as mean ± SEM.

We next investigated the neuroprotective potential of the SWELL1 inhibitor Dicumarol in vivo. To establish a suitable treatment regime, we first assessed the concentration of Dicumarol in plasma and brain following intraperitoneal (i.p.) administration. We found that Dicumarol levels in the brain reached ≈3 µmol kg^−1^, which was less than 3% of that in the plasma (Figure S6A, Supporting Information), indicating poor blood‐brain barrier (BBB) penetration. Therefore, we directly infused Dicumarol intracerebrally, enabling effective perfusion of large brain volumes independent of BBB penetration (**Figure** **5**A). Indeed, Dicumarol concentration reached ≈45 µmol kg^−1^ (*n* = 5 mice) after intracerebral delivery, nearly completely inhibiting the SWELL1 channel in vitro. We then conducted a proof of principle study to evaluate the effect of Dicumarol on ischemic stroke. Mice injected with Dicumarol 20 min before surgery showed significantly smaller infarct volumes and reduced hemispheric swelling 1 day after tMCAO compared to vehicle‐treated mice (Figure 5B–D). Additionally, Dicumarol‐treated mice exhibited improved neurological deficit scores (Figure 5E). These findings are consistent with the inhibitory effects of Dicumarol on the SWELL1 channel observed in vitro and in acute brain slices (Figure 4), suggesting that Dicumarol provides neuroprotection against experimental stroke in vivo.

Originally discovered from molding sweet‐clover hay, Dicumarol is the prototype of the hydroxycoumarin anticoagulant drug class that inhibits vitamin K epoxide reductase, thereby depleting stores of vitamin K and preventing blood clotting.^[^ ^42^ ^]^ Warfarin, another anticoagulant of the same family,^[^ ^43^ ^]^ failed to inhibit hypotonicity‐induced SWELL1 currents in astrocytes (Figure S6B–D, Supporting Information), suggesting that Dicumarol blocks SWELL1 channel independent of its effect on vitamin K epoxide reductase. Importantly, unlike Dicumarol, Warfarin also failed to provide neuroprotection in the experimental stroke mouse model (Figure 5B–E). These results indicate that the neuroprotective effect of Dicumarol is independent of its ability to deplete vitamin K stores and its anticoagulant activity. To assess the potential hemorrhagic side effects of Dicumarol treatment, we measured the hemoglobin concentration and found comparable levels of hemoglobin in the stroke hemispheres injected with Dicumarol or DMSO (Figure S6E, Supporting Information). This suggests that Dicumarol treatment in our experiments did not lead to excessive hemorrhage.

**Figure 6:**
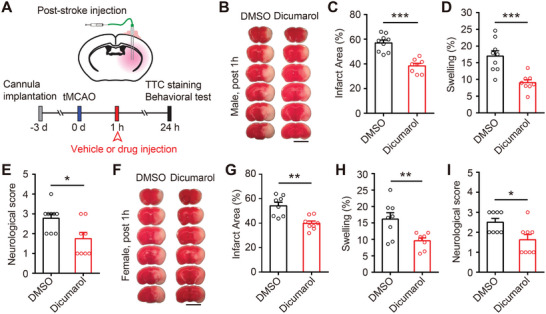
Dicumarol treatment after ischemia also reduces brain damage in experimental stroke in both male and female mice. A) Experimental design of the ischemic stroke study. Cannulas were implanted in the brains of mice 3 days before tMCAO surgery. 10 µL DMSO (1%) or Dicumarol (100 µm in 1% DMSO) was infused into the brain at 1 h after tMCAO. Neurological deficit scores were evaluated 1 day after tMCAO, and brain samples were then collected for TTC staining. B) Representative images of TTC staining 1 day after tMCAO in male mice. Scale bar, 5 mm. C,D) Quantification of total infarct area volume C) and hemispheric swelling D) in (B). *n* = 8‐–9 mice for each group. Student's *t*‐tests, ^***^ *p *< 0.001. E) Quantification of neurological deficit scores in male mice. *n* = 8–9 mice for each group. Mann‐Whitney test, ^*^ *p *< 0.05. F) Representative images of TTC staining 1 day after tMCAO in female mice. Scale bar, 5 mm. G,H) Quantification of total infarct area volume G) and hemispheric swelling H) in (F). *n* = 8 mice for each group. Student's *t*‐tests, ^**^ *p* < 0.01. I) Quantification of neurological deficit scores in female mice. *n* = 8 mice for each group. Mann–Whitney test, ^*^ *p *< 0.05. Data are reported as mean ± SEM.

To determine the extent of neuroprotection provided by Dicumarol, we evaluated neurological deficit scores over one week and measured brain infarct volume at day 7 post‐tMCAO with TTC (Figure S7A, Supporting Information).^[^ ^44^, ^45^ ^]^ Dicumarol treatment resulted in a persistent reduction in infarct sizes compared to the vehicle‐treated group (Figure S7B,C, Supporting Information). Consistently, mice with Dicumarol administration exhibited a decrease in neurological deficit scores over 7 days post‐stroke (Figure S7D, Supporting Information). Dicumarol thus produced both early and sustained improvement in stroke‐associated brain damage and neurological functions. To further evaluate its therapeutic potential, we investigated whether Dicumarol treatment initiated after the experimental stroke onset also provides neuroprotection. For this purpose, we implanted a cannula into the brain to deliver the drug after tMCAO (**Figure** **6**A). Administration of Dicumarol through the cannula 1 h after ischemia induction (Figure 6B‐E) significantly reduced infarct volumes, hemispheric swelling, and neurological deficit scores, although to a lesser extent compared to pre‐stroke treatment. These results suggest that Dicumarol administration, whether before or after tMCAO, mitigates ischemic brain damage. To determine if sex is a biological variable for the neuroprotective effect of Dicumarol, we performed tMCAO surgery in female mice. Similar to observations in male mice, Dicumarol treatment in female mice after tMCAO also resulted in significantly smaller infarct volumes and less hemispheric swelling with better neurological outcomes compared to vehicle‐treated controls (Figure 6F–I). These results collectively demonstrate that Dicumarol exerts broad neuroprotection in the experimental stroke mouse model, irrespective of whether treatment is initiated before or after the onset of injury.

### 2.6 A Screen for Dicumarol Derivatives Identifies Additional SWELL1 Channel Inhibitors

**Figure 7:**
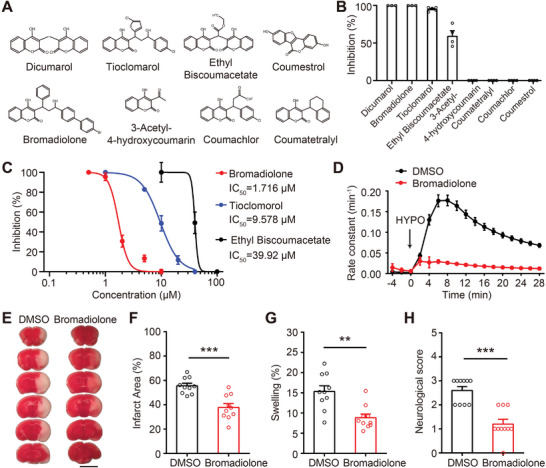
Screening of Dicumarol derivatives identifies Bromadiolone as another potent SWELL1 channel inhibitor with neuroprotective effects. A) Chemical structures of seven Dicumarol derivates selected for examining the inhibitory effect on the SWELL channel. B) SWELL1 currents were inhibited by Bromadiolone, Tioclomarol, and Ethyl Biscoumacetate. *n* = 3–5 HeLa cells for each group. C) Dose‐response curves of three Dicumarol derivates. Each data point represents the mean ± SEM (*n* = 3–5 HeLa cells). D) Time course of the rate constant for swelling‐induced [^3^H] taurine efflux after the exposure of hypotonic solution (indicated by the arrow) in HeLa cells treated with DMSO or Bromadiolone. Data represent three independent experiments. E) Representative images of TTC staining 1 day after tMCAO. 10 µl DMSO (1%) or Bromadiolone (15 µm in 1% DMSO) was infused into the brain 20 min before tMCAO surgery in male mice. Scale bar, 5 mm. F,G) Quantification of total infarct area volume F) and hemispheric swelling G). *n* = 10 mice for each group. Student's *t*‐test, ^**^ *p *< 0.01 and ^***^ *p *< 0.001. H) Quantification of neurological deficit scores 1 day after tMCAO. *n* = 10 mice for each group. Mann‐Whitney test, ^***^ *p* < 0.001. Data are reported as mean ± SEM.

To identify additional SWELL1 channel inhibitors, we screened seven hydroxycoumarin anticoagulants from the same family as Dicumarol (**Figure** **7**A). Patch‐clamp recordings revealed that three compounds potently inhibited hypotonicity‐induced SWELL1 currents (Figure 7B). This suggests that SWELL1 channel inhibitors are commonly found among Dicumarol‐related compounds. These newly identified inhibitors also blocked SWELL1 currents in a dose‐dependent manner, with IC_50_ values ranging from 1.7 to 39.9 µm (Figure 7C). Notably, Bromadiolone exhibited exceptional potency with an IC_50_ of 1.7  µm, surpassing Dicumarol. Besides Cl^−^ currents, Bromadiolone also robustly inhibited SWELL1 channel‐mediated amino acid/neurotransmitter release, as evidenced by the suppression of [^3^H]‐labeled taurine efflux upon hypotonic stimulation (Figure 7D). To test whether Bromadiolone provides neuroprotection in vivo, we injected Bromadiolone into the brain prior to stroke induction and evaluated brain infarct volumes and hemispheric swelling 1 day post‐tMCAO (Figure 7E–G). Consistently, Bromadiolone‐treated mice exhibited reduced infarct sizes and less hemispheric swelling compared to vehicle‐treated mice (Figure 7E–G). Moreover, behavioral assessments showed improved scores in Bromadiolone‐treated mice relative to controls (Figure 7H). Together with the findings from Dicumarol, these results underscore the therapeutic promise of targeting the SWELL1 channel as a potential strategy for treating ischemic stroke.

## 3 Discussion

**Figure 8:**
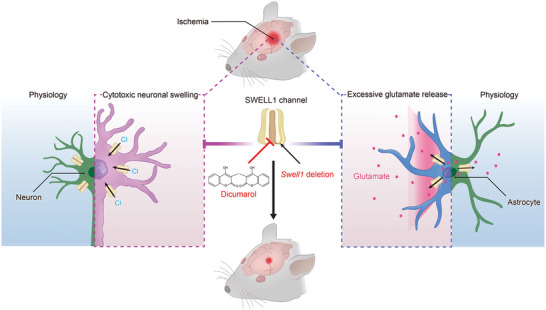
Model for the pleiotropic effects of the SWELL1 channel in ischemic stroke pathogenesis. The SWELL1 channel plays a critical role in ischemic stroke pathology by promoting cytotoxic neuronal swelling and glutamate excitotoxicity. This dual mechanism contributes significantly to ischemic damage in the brain. Dicumarol, a novel SWELL1 channel blocker and FDA‐approved drug, effectively inhibits the two pathways, demonstrating robust neuroprotective effects in experimental stroke models.

Current therapies for ischemic stroke are limited to tissue plasminogen activators and endovascular thrombectomy.^[^ ^6^, ^46^ ^]^ Clinical trials for stroke focusing on single disease mechanisms are often considered insufficient to suppress the ischemic cascades.^[^ ^7^ ^]^ Here, we report that the SWELL1 channel plays a dual role in the pathogenesis of ischemic stroke, representing a promising target for this devastating disease. Ischemia upregulates SWELL1 expression in both neurons and astrocytes of the peri‐infract areas, leading to pathological consequences, including cytotoxic neuronal swelling and glutamate excitotoxicity. Importantly, Dicumarol, a clinically available drug, potently inhibits the SWELL1 channel and provides robust and persistent neuroprotection in an experimental stroke mouse model (**Figure** **8**). Our data provide a strong rationale for pursuing the SWELL1 channel as a therapeutical target for the treatment of ischemic brain damage.

In this study, we demonstrate that SWELL1 is required for an important yet under‐explored stroke injury mechanism: cytotoxic neuronal swelling. Neuronal swelling involves an influx of both Na^+^ and Cl^−^, but the pathways for Cl^−^ influx have remained elusive.^[^ ^9^ ^]^ A previous study identified the ion exchanger SLC26A11 acting as a voltage‐gated Cl^−^ channel that partially mediates Cl^−^ entry during neuronal swelling.^[^ ^10^ ^]^ However, substantial swelling still occurred after efficient knockdown of SLC26A11, suggesting that additional mechanisms of Cl^−^ influx exist. Interestingly, treatment with DIDS, a non‐specific blocker inhibiting both SLC26A11 and VRAC, effectively abolished Cl^−^ influx and neuronal swelling induced by intracellular Na^+^ accumulation. Here, we employed a comprehensive set of approaches, including specific deletion of *Swell1* in pyramidal neurons, RNAscope in situ hybridization, whole‐cell patch‐clamp electrophysiology, live‐cell imaging, and cell death assays. We showed that intracellular hypertonicity with increased NaCl following ischemia activates SWELL1 currents, which contributes to the subsequent neuronal swelling and cell death. This new mechanism may explain the previous observation that deleting SWELL1 in both neurons and astrocytes provided a more robust neuroprotection than deleting in astrocytes alone.^[^ ^21^, ^22^, ^23^ ^]^

Tremendous efforts have been made to inhibit deleterious NMDAR signaling for treating ischemic stroke. Nevertheless, NMDAR antagonists have proven largely unsuccessful as neuroprotectants in clinical trials, as they also affect the physiological functions of NMDARs essential for neuronal survival.^[^ ^47^, ^48^ ^]^ Targeting excessive glutamate release upstream of NMDAR activation may be a preferable approach to mitigating glutamate excitotoxicity without interfering with the physiological function of NMDARs.^[^ ^49^ ^]^ A major hurdle for this strategy is identifying specific pathways of glutamate release from astrocytes in ischemic stroke, which may include ionotropic purinergic receptors, cystine‐glutamate antiporter, and hemichannels.^[^ ^50^, ^51^, ^52^, ^53^, ^54^ ^]^ Our study revealed that the SWELL1 channel in astrocytes plays an important role in pathological glutamate release after ischemia. By examining the detailed expression pattern of SWELL1 in different regions after tMCAO, we found that astrocytic SWELL1 expression is upregulated in the peri‐infarct region. More importantly, the SIC frequency and tonic NMDAR currents, as indicators of glutamate release, were also increased after experimental stroke. These changes were reduced by both deletion of astrocytic *Swell1* and pharmacological inhibition of the SWELL1 channel. Therefore, our results establish a direct mechanistic relationship between the SWELL1 channel and ischemia‐induced glutamate excitotoxicity.

Previous studies with non‐specific channel blockers have implicated VRAC in ischemic stroke, however, definitive evidence for its involvement was missing due to the lack of molecular identity. In the current study, we provide direct genetic evidence that SWELL1, the essential subunit of VRAC, is required for both cytotoxic neuronal swelling and glutamate excitotoxicity. Based on these results, we further validated the therapeutic potential of inhibiting the SWELL1 channel by repurposing an existing drug, Dicumarol. We demonstrated a previously unidentified role of Dicumarol in inhibiting the SWELL1 channel and providing neuroprotection in an experimental stroke mouse model. SWELL1 constitutive KO mice are embryonic lethal,^[^ ^23^ ^]^ and brain‐wide SWELL1 deletion also results in growth retardation and early mortality. Thus, it's not feasible to test Dicumarol in these KO mice and directly validate its SWELL1 channel‐on‐target activity. Instead, taking advantage of the large family of hydroxycoumarin anticoagulant drugs, we showed that the closely related Warfarin does not inhibit the SWELL1 channel and has no neuroprotection, while Bromadiolone inhibits the channel and exhibits neuroprotective effects. Thus, this alternative approach suggests the SWELL1‐on‐target activity of Dicumarol for ischemic stroke in vivo, although we cannot exclude the possibility that it may also engage additional neuroprotective pathways.

Dicumarol has been used as an anticoagulant to treat deep venous thrombosis and atrial fibrillation.^[^ ^42^ ^]^ Interestingly, current guidelines also recommend anticoagulation agents for ischemic stroke prevention in high‐risk patients with atrial fibrillation.^[^ ^55^ ^]^ Clinical trials on the prevention of stroke among these patients have consistently shown a benefit associated with anticoagulation therapy.^[^ ^56^ ^]^ As an FDA‐approved drug, Dicumarol's pharmacology, pharmacokinetics, and toxicity are well studied in human and non‐human lab animals. Therefore, the use of Dicumarol among stroke patients with atrial fibrillation, who often have more severe strokes and poorer neurological outcomes, may provide additive benefits from both its anticoagulant effects and SWELL1 channel blockade. One key limitation of Dicumarol is its poor BBB penetration. One potential solution is to directly deliver the drug into the ischemic brain region,^[^ ^57^, ^58^ ^]^ as we performed in the animal model. This approach also minimizes the perceived risk of bleeding, although we did not observe this in the mice study. Alternatively, our identification of Dicumarol and Bromadiolone provides a foundation for future medicinal chemistry optimizations to improve their potency and pharmacological properties (e.g. BBB permeability). This will pave the way for the future translation of SWELL1‐targeting therapies for ischemic stroke and other diseases associated with abnormal VRAC channel activity.

## 4 Experimental Section

### Mice

All procedures related to animal care and treatment were approved by the Johns Hopkins University Animal Care and Use Committee and met the guidelines of the National Institute of Health Guide for the Care and Use of Laboratory Animals (No. MO22M17). All animals were group housed in a standard 12 h light/12 h dark cycle with ad libitum access to food and water. Both male and female animals were used for all experiments. KO mice were generated by using a *Cre/loxP* recombination system. The homozygous *Swell1^F/F^* mice were previously described^[^ ^21^ ^]^ and were maintained in the laboratory. WT C57BL/6J, *mGFAP*‐cre (B6. Cg‐Tg(Gfap‐cre)77.6Mvs/2J), and *Ai14* (B6.Cg‐Gt(ROSA)26Sor^tm14(CAG‐tdTomato)Hze^/J) were purchased from The Jackson Laboratory. *NEX*‐cre was a gift from Klaus‐Armin Nave^[^ ^29^ ^]^.

### Antibodies and Chemicals

The antibodies and chemicals used in this study are listed in Table S1 (Supporting Information).

### Cell Culture and Transfection

HeLa cells and HEK293T cells were maintained in Dulbecco's modified Eagle's medium supplemented with 10% fetal bovine serum (FBS) and 1% penicillin/streptomycin (P/S) at 37 °C in a humidified 95% CO_2_ incubator. *SWELL1* KO HeLa cells were generated by CRISPR/Cas9‐mediated gene deletion as described previously.^[^ ^21^ ^]^ Cells were seeded on 12 mm diameter poly‐D‐lysine (PDL) coated glass coverslips (BD) in 24‐well dishes and recorded 1 day later.

HEK293T cells were co‐transfected with EGFP and GluR1‐L497Y plasmids using Lipofectamine 2000 (Invitrogen) 1 day before the sniffer patch recording. On the day of sniffer patch experiments, HEK293T cells expressing GluR1‐L497Y were dissociated, triturated, and seeded onto coverslips containing primary astrocytes. Cells were not cultured past 20 passages.

To culture primary astrocytes, cortices from P0‐P1 newborn pups were dissected in ice‐cold Hanks’ Balanced Salt Solution and digested in 0.25% trypsin at 37 °C for 20 min. Cells were dissociated by triturating 15–20 times in the culture media (Minimum Essential Media supplemented with 10% FBS and 1% P/S) and plated into a culture flask for 7–10 days. The purity of astrocyte cultures was > 95% as routinely confirmed with GFAP immunostaining. For astrocyte recording, cells were digested and plated onto PDL‐coated coverslips at least 1 day before recording.

### Cell Patch‐Clamp Electrophysiology

For hypotonicity‐activated SWELL1 current recordings, HeLa cells or primary astrocytes were whole‐cell patched in an isotonic bath solution containing (in mm): 145 NaCl, 2 KCl, 1 MgCl_2_, 1 CaCl_2_, 10 HEPES, and 10 Glucose (pH 7.4 adjusted with NaOH and osmolality was 300–310 mOsm kg^−1^), then perfused with a hypotonic solution containing (in mm): 95 NaCl, 2 KCl, 1 MgCl_2_, 1 CaCl_2_, 10 HEPES, and 10 Glucose (pH 7.4 adjusted with NaOH and osmolality was 210–220 mOsm kg^−1^). Recording electrodes (2–4 MΩ) were filled with a standard internal solution containing (mm):133 CsCl, 10 HEPES, 4 Mg‐ATP, 0.5 Na_3_‐GTP, 2 CaCl_2_, and 5 EGTA (pH adjusted to 7.2 with CsOH and osmolality was 290–300 mOsm kg^−1^). For increasing intracellular Na^+^‐activated SWELL1 current recordings, HeLa cells were whole‐cell patched in the same isotonic bath solution. Recording electrodes were filled with a standard internal solution plus additional NaCl. For the VRAC time course, constant voltage ramps (5 s interval, 500 ms duration) were applied from a holding potential of 0 to ±100 mV.

### Sniffer Patch Recording

Cultured primary astrocytes were seeded sparsely on PDL‐coated coverslips and used as the source cells. HEK293T cells co‐transfected with GluR1‐L497Y and EGFP were the sensor cells. 12–18 h after transfection, HEK293T cells were reseeded onto donor cells for recording. To activate SWELL1 channel in astrocytes, recording electrodes (2‐4 MΩ) were filled with a hypertonic internal solution containing (mm): 133 CsCl, 10 HEPES, 4 Mg‐ATP, 0.5 Na_3_‐GTP, 2 CaCl_2_, 5 EGTA, 100 mannitol, and 5 glutamate (pH adjusted to 7.2 with CsOH and osmolality was 400–410 mOsm kg^−1^). For HEK293T cell recording, the same internal solution was used without mannitol and glutamate. The external solution contained (in mm): 145 NaCl, 10 HEPES, 2 KCl, 2 CaCl_2_, 1 MgCl_2_, and 10 glucose (pH adjusted to pH 7.3 with NaOH and osmolality adjusted to 300–310 mOsm kg^−1^). Dicumarol was added to the bath to inhibit SWELL1 currents. All the cells were held at −60 mV using a Multiclamp 700B amplifier, and data were acquired with pClamp 10.7 software (Molecular Devices).

### Taurine Efflux Assay

Taurine efflux assay was performed as previously described^17^. HeLa cells in 24‐well plates were loaded with culture medium containing [^3^H] taurine (1 µCi mL^−1^) at 37 °C for 2 h. The cells were washed 5 times with isotonic solution (in mm): 145 NaCl, 2 KCl, 1 MgCl_2_, 1 CaCl_2_, 10 HEPES, and 10 Glucose (pH 7.4 adjusted with NaOH and osmolality was 300–310 mOsm kg^−1^). The experiment was performed by removal/addition of 0.5 mL aliquots of solutions at 2 min intervals. The efflux was initiated by aspiration of the isotonic solution followed by the addition of hypotonic solution (in mm): 95 NaCl, 2 KCl, 1 MgCl_2_, 1 CaCl_2_, 10 HEPES, and 10 Glucose (pH 7.4 adjusted with NaOH and osmolality was 210–220 mOsm kg^−1^). The cells were lysed at the end of the experiment with 0.5 m NaOH. The total ^3^H activity in the cells was estimated as the sum of ^3^H activity in all efflux samples and the final cell lysates using a Beckman Coulter LS 6500 liquid scintillation counter. The natural logarithm to the fraction of ^3^H activity remaining in the cells was plotted versus time, and the rate constant for the taurine efflux (min^−1^) at each time point was subsequently estimated as the negative slope of the curve between the time point and the proceeding time point.

### RNA and Protein Isolation

The samples for quantitative real‐time PCR and immunoblots were prepared as previously described.^[^ ^59^ ^]^ According to previous publications,^[^ ^45^ ^]^ there is a small infarction in the striatum at 1.5 h after occlusion. Then the infarct core is enlarged and still limited to the striatum by 6 h, and the penumbra begins to be seen as pink TTC staining around the infarct core. At 12–24 h, almost all of the striatum and large areas of the cortex are infarcted. Therefore, RNA and protein isolation and penumbra area electrophysiology 8 h after occlusion were choosed to perform. For the RNA and protein isolation, brains were removed and cut into six 1 mm serial coronal sections using a vibratome (VT‐1200S, Leica). The sections were stained with 1.5% 2,3,5‐triphenyltetrazolium chloride (TTC) in PBS for 3 min at 37 °C. The uninvolved hemisphere, peri‐infarct region, and infarct core were then dissected from TTC‐stained brain slices. Total RNA was extracted with ice‐cold TRIzol reagent (Invitrogen) and used for real‐time PCR analysis. For protein isolation, tissue samples were homogenized in ice‐cold RIPA buffer (150 mm NaCl, 50 mm Tris, pH 8.0, 0.1% SDS, 0.5% deoxycholate, 1% Nonidet P‐40, 1 mm phenylmethanesulfonyl fluoride and protease inhibitor mixtures), and lysed on ice for 30 min. The mixture was centrifuged at 12 000 rpm for 20 min at 4 °C. Supernatants were isolated and protein concentration was measured using a Pierce BCA protein assay kit and analyzed by western blotting.

### Quantitative Real‐Time PCR

Total RNA was reverse‐transcribed into cDNA by random primers, which was then used as templates for real‐time PCR with PerfeCTa FastMix II, low ROX (Quanta Biosciences). The reaction was run in the QuantStudio 6 Flex Real‐time PCR system using 1 µL of the cDNA in a 10 µL reaction according to the manufacturer's instruction in triplicate. Results were analyzed using the 2^−∆∆CT^ method, and normalized to *β‐actin* mRNA. Mouse *Swell1* primer set (Mm.PT.58.12679347) and *β‐actin* primer set (Mm.PT.58.33257376.gs) were purchased from Integrated DNA Technologies.

### Western Blot

Samples were separated by 10% SDS/PAGE and transferred onto nitrocellulose membranes, which were incubated in the PBS buffer containing 0.1% Tween‐20 and 5% non‐fat milk for 1 h at room temperature (RT) before the addition of primary antibody (anti‐SWELL1; 1:1000; from a rabbit immunized with SWELL1 C‐terminal peptide antigen) for incubation overnight at 4 °C. After wash, the membranes were incubated with HRP‐conjugated secondary antibody (Thermo Scientific) for 1 h at RT. Immunoreactive bands were carried out using enhanced chemiluminescence, and the signals were collected by the ChemiDoc Touch Imaging system before the analysis with ImageJ (NIH).

### RNAscope In Situ Hybridization

RNAscope In situ hybridization was performed as previously described.^[^ ^21^ ^]^ Anesthetized mice were perfused transcardially with PBS, followed by 4% paraformaldehyde (PFA) in PBS. Brains were removed and post‐fixed in 4% PFA at 4 °C overnight, dehydrated, and embedded in OCT (Tissue‐Tek) before being sectioned into tissue slices (12 µm thick). RNAscope Multiplex Fluorescent Reagent Kit v.2 (ACD, Advanced Cell Diagnostics) was used following the manufacturer's manual for the fixed frozen tissues. Probes targeting *Swell1* (#458371) were purchased from ACD. TSA Cyanine 3 (#NEL744) or TSA Cyanine 5 (#NEL745) was used for developing the fluorescence signal. After RNAscope in situ hybridization, sections underwent immunostaining with Alexa Fluor‐conjugated anti‐Iba1 (1:1000; FUJIFILM) for 1 h at RT. After washing with PBS 3 times for 5 min, images were taken on a Zeiss LSM 900 confocal microscope.

### Brain Slice Preparation for Recording, Imaging, and Cell Death Assays

Mice were anesthetized with the inhalation anesthetic isoflurane and then perfused with the ice‐cold oxygenated cutting solution. The brain was removed rapidly and immersed in the ice‐cold choline‐based cutting solution containing (in mm): 110 choline chloride, 7 MgCl_2_, 2.5 KCl, 0.5 CaCl_2_, 1.3 NaH_2_PO_4_, 25 NaHCO_3_, 20 glucose, saturated with 95% O_2_ and 5% CO_2_. Coronal brain slices (250 µm) were cut in the cutting solution using a vibratome (VT‐1200S, Leica) and transferred to artificial cerebrospinal fluid (aCSF) containing (in mM): 125 NaCl, 2.5 KCl, 2.5 CaCl_2_, 1.3 MgCl_2_, 1.3 NaH_2_PO_4_, 26 NaHCO_3_, 10 glucose, saturated with 95% O_2_ and 5% CO_2_. The slices were allowed to recover for 40 min at 32 °C and then at RT for at least 1 h before recording or imaging experiments.

### Acute Brain Slice Recording

Mice were sacrificed at 8 h after the tMACO surgery. All recordings were made at RT in a submerged recording chamber with constant aCSF perfusion. Neurons were visualized under an upright microscope (BX51WI, Olympus) with infrared optics. Recording pipettes were pulled by a micropipette puller (P‐1000, Sutter instrument). Recordings were made with MultiClamp 700B amplifier and a 1550B digitizer (Molecular Device). Data acquisition was performed with pClamp 10.7 software (Molecular Device), filtered at 1 kHz, and digitized at 10 kHz. In all experiments, the series resistance (*R*_s_) was monitored throughout the recording and controlled below 20 MΩ with no compensation. Data were discarded when the series resistance varied by ≥20%.

For SIC recordings, whole‐cell recordings from cortical layer 2/3 pyramidal neurons were performed with an internal solution containing (in mM): 125 K‐gluconate, 15 KCl, 10 HEPES, 1 MgCl_2_, 4 Mg‐ATP, 0.3 Na_3_‐GTP, 10 phosphocreatine, and 0.2 EGTA (pH 7.2, osmolality 290–300 mOsm kg^−1^). Neurons were held at −70 mV in the presence of 1 µm TTX, 100 µm picrotoxin, 20 µm DNQX, and 10 µm D‐serine. When the baseline was stable, Mg^2+^ free aCSF was applied for 10 min.

For tonic NMDAR current recordings, cortical layer 2/3 pyramidal neurons were held at +40 mV in normal aCSF with an internal solution containing (in mm): 110 Cs methylsulfate, 20 TEA‐Cl, 15 CsCl, 4 ATP‐Mg, 0.3 Na_3_‐GTP, 0.5 EGTA, 10 HEPES, 4.0 QX‐314, and 1.0 spermine (pH 7.2, osmolality 290–300 mOsm kg^−1^). Recordings were performed in the presence of 1 µm TTX and 20 µm DNQX. After the baseline was stable, tonic NMDAR current was observed by bath application of D‐AP5.

For increasing intracellular Na^+^‐induced SWELL1 current recordings, hippocampal CA1 pyramidal neurons were held at −70 mV in the presence of 1 µm TTX, 100 µm picrotoxin, 20 µm DNQX and 50 µm D‐AP5. The internal solution was the same as the SIC recordings plus an additional 40 mm NaCl. Dicumarol was bath applied at 15–20 min after the whole‐cell configuration was made.

### Biocytin Labeling

To mark the position of recorded neurons in brain slices from tMCAO mice, the recording pipettes were filled with 0.1% biocytin (Sigma). After recording, pipettes were removed slowly and the brain slices were fixed in 4% PFA overnight, permeabilized with 0.2% Triton X‐100 and 1% BSA in PBS for 1 h at RT, and then incubated with fluorophore‐conjugated streptavidin (1:1000; Invitrogen) for 2 h. After washing with PBS solution 3 times for 5 min, confocal images were obtained with a Zeiss LSM 900 imaging system.

### Live Cell Imaging in Acute Brain Slices

Mice (P15‐17) were injected with pAAV‐CaMKIIα‐EGFP (Addgene) before imaging. Briefly, a Hamilton syringe with a 33‐gauge needle was loaded with 0.6 µL of the virus (3 × 10¹^2^ vg mL^−1^) and fixed to the stereotaxic frame. The needle was put in the cortex with the following coordinates: 1.2 mm anterior‐posterior, ±1.2 mm lateral, and 0.7 mm deep from the bregma. The solution was infused with a 0.2 µL min^−1^ rate using an injection pump (KD Scientific). 10–14 days after the virus injection, brains were removed and slices were prepared as described in the brain slice preparation section above. Imaging of the brain slices was performed at RT in a submerged chamber with constant perfusion of aCSF (125 mm NaCl, 2.5 mm KCl, 2.5 mm CaCl_2_, 1.3 mm MgCl_2_, 1.3 mm NaH_2_PO_4_, 26 mm NaHCO_3_, 10 mm glucose) with a cocktail of ligand‐gated and voltage‐gated ion channel inhibitors (100 µm picrotoxin, 20 µm DNQX, 30 µm Cadmium, 100 µm D‐AP5), saturated with 95% O_2_ and 5% CO_2_. For imaging under low Cl^−^ conditions, the aCSF was made by replacing NaCl with equimolar amounts of Na‐gluconate. 50 µm veratridine was then applied to brain slices for 10 min. The images were taken on a Zeiss LSM 900 confocal microscope with a 40x/1.2 numerical aperture objective lens and were further analyzed using ImageJ.

### Assessment of Cell Death in Acute Brain Slices

Cell death was assessed by propidium iodide (PI) uptake in mouse hippocampal slices, which were prepared as described above. Hippocampal slices were pretreated at RT for 10 min with a cocktail of ligand‐gated and voltage‐gated ion channel inhibitors (100 µm picrotoxin, 20 µm DNQX, 30 µm Cadmium, 100 µm D‐AP5), saturated with 95% O_2_ and 5% CO_2_. 50 µm veratridine was then applied to brain slices for 10 min. After washing, the brain slices were transferred to an incubation chamber and further incubated for 1 h. The brain slices were then stained with PI (10 µg mL^−1^) and Hoechst 33342 (1 µg mL^−1^) at RT for 10 min, and fixed with 4% PFA for 15 min. Live cells have intact membranes that exclude PI which penetrates the damaged permeable membranes of non‐viable cells. Cells stained with PI in the CA1 pyramidal cells were counted. Cell mortality was expressed as the ratio of PI‐positive cells to total cell number determined by counting cells stained with Hoechst 33342.

### Intracerebral Drug Delivery

Male and female C57BL/6J mice (6 weeks, ≈20 g), randomly assigned to different groups, received intracerebral injections of 10 µL of Dicumarol (100 µm in 1% DMSO), Bromadiolone (15 µm in 1% DMSO), Warfarin (100 µm in 1% DMSO), or Vehicle (1% DMSO) either before or after tMCAO. The dosing and concentration for Dicumarol and Bromadiolone were determined according to the LC/MS analytic method and a previous study of non‐specific VRAC inhibitors delivered to the brain with the same route, and were modified based on the IC_50_ of these inhibitors.^[^ ^15^ ^]^ For the pre‐stroke drug delivery, mice were anesthetized with isoflurane and placed into a stereotactic apparatus. The body temperature was maintained at 37 °C during surgery with a homeothermic monitoring system (RWD). A Hamilton syringe with a 26‐gauge needle was loaded with 10 µL of each solution and fixed to the stereotaxic frame. The needle was put in the right hemisphere of the brain with the following coordinates: −0.3 mm anterior‐posterior, −2.5 mm lateral, and 3.5 mm deep from the bregma. The solution was infused with a 0.5 µL min^−1^ rate using an injection pump (KD Scientific), followed by immediate tMCAO surgery. For the post‐stroke drug delivery, a 26‐gauge stainless steel guide cannula (RWD) was implanted in the right hemisphere of the brain (−0.3 mm anterior‐posterior, −2.5 mm lateral, and 3.5 mm deep from the bregma) and was fixed to the skull with dental cement. After 3 days of recovery, the mice were subjected to tMCAO surgery followed by drug delivery to the brain slowly by using a microsyringe pump (10 µL, 0.5 µL min^−1^ rate) which was connected to the infusion cannula by a PE20 tube.

### Dicumarol Measurement by LC/MS

Mouse plasma and brain samples were collected 30 min after intraperitoneal injection of 10 mg kg^−1^ Dicumarol. The samples were stored at −80 °C until analysis. To quantify Dicumarol, methanol containing 0.5 µm losartan as an internal standard was added to 5 µL µL^−1^ plasma or 5 µL mg^−1^ brain in microcentrifuge tubes. The brain was homogenized using a Spex Geno/Grinder® with stainless steel beads for 3 min at 1750 RPM. Plasma and brain homogenates from untreated animals were spiked with Dicumarol from 100 to 0.01 nmol mL^−1^ plasma or nmol/g brain by serial dilution to generate standard curves. Plasma and brain homogenates were then vortexed and centrifuged (16,000 x g for 5 min at 4 °C) to precipitate proteins. Supernatants were transferred to a 96 well plate and 0.5 µL was injected on an Ultimate 3000 LC coupled to a QExactive mass spectrometer (Thermo Fisher Scientific Inc., Waltham MA). Samples were separated on an Agilent EclipsePlus C18 RRHD (1.8 µm) 2.1 × 100 mm column. The mobile phase consisted of water + 0.1% formic acid (A), and acetonitrile + 0.1% formic acid (B). Separation was achieved at a flow rate of 0.4 mL min^−1^ using a gradient run, from 40/60 (A/B), ramped linearly 0/100 (A/B) over 1 min, maintaining at 0/100 (A/B) for 1.5 min, and then re‐equilibrating in starting conditions for 1 min. Samples were introduced to the source through heated ion spray with the capillary temperature setting at 350 °C, spray voltage of 2 kV, and S‐Lens RF of 35. Nitrogen was used as the sheath and auxiliary gas with the settings of 30 and 3 arbitrary units respectively. Transitions of 335.0561 to 117.0345 and 161.0244 (dicoumarol) and 423.1695 to 127.0069 and 179.0867 (losartan) were acquired with collision energies settings of 15 and 40 CE respectively. Peaks were quantified with Xcalibur software.

### Transient Middle Cerebral Artery Occlusion (tMCAO)

The tMCAO surgery and downstream histological and behavioral assays were performed by an investigator blinded to treatment conditions. The mice were anesthetized with isoflurane, and body temperature was maintained at 37 °C during surgery with a homeothermic monitoring system (RWD). Ischemia was induced by introducing a 0.19 mm silicone‐coated monofilament (7‐0; Doccol) from the external carotid artery into the internal carotid artery and advancing it into the right MCA origin. Reperfusion was performed 45 min after occlusion by removing the occluding suture from the MCA as described previously.^[^ ^22^ ^]^ The mortality rate after surgery is ≈10% and similar among different treatment groups. The dead mice were excluded from further characterization. Neurological function was evaluated as described previously in a double‐blind fashion with a scale of 0–5: 0, no neurological deficit; 1, failed to extend right forepaw; 2, circled to the right; 3, fell to the right; 4, could not walk spontaneously and; 5, death.^[^ ^21^ ^]^ After neurological evaluation, brains were removed and cut into six 1 mm serial coronal sections using a vibratome (VT‐1200S, Leica). The sections were stained with 1.5% TTC in PBS for 5 min at 37 °C, followed by fixation in 4% PFA for 15 min. The TTC‐stained sections were scanned and digitized. Using ImageJ, the areas of TTC negative and the ipsilateral hemisphere were measured. The percent volume of infarct was calculated as: (the sum area of infarct/the sum area of the ipsilateral hemisphere). The percent swelling was calculated as: ((area of ipsilateral hemisphere − area of contralateral hemisphere)/area of contralateral hemisphere).

### Hemoglobin Measurements

Mice were anesthetized with the inhalation anesthetic isoflurane and then perfused with PBS solution containing 0.16 mg mL^−1^ heparin to remove extraneous red blood cells. The ischemic hemisphere was harvested and homogenized in 1 mL of PBS solution before centrifugation at 10,000 x g for 15 min at 4 °C. The supernatant was collected, and hemoglobin concentration was measured using a Hemoglobin Colorimetric Assay kit according to the manufacturer's instructions (Caymen Chemical Company, Ann Arbor, MI).

### Statistical Analyses

All data points or bars are presented as mean ± SEM. GraphPad Prism 8.0 software (San Diego, CA) was used for all statistical analyses. Prior to each test, the Gaussian distribution of the data was assessed using the Shapiro–Wilk or Kolmogorov‐Smirnov normality test to determine whether the data were normally distributed. A parametric test (two‐tailed Student's *t*‐test for two groups or one‐way ANOVA with Bonferroni post hoc test for more than two groups) was used for normally distributed data. A non‐parametric test (Mann–Whitney for unpaired two groups or Kruskal–Wallis test followed by Dunn's post hoc test for three or more groups) was used for data not normally distributed. Two‐way ANOVA followed by Bonferroni's post hoc test was used to analyze studies that have two factors. The significance level was set at *p* < 0.05. Experimental and control animals were randomized throughout the study. Investigators were blinded to the allocation of groups and outcome assessment for all experiments.

## Conflict of Interest

The authors declare no conflict of interest.

## Author Contributions

J.C. performed cultured cell patch‐clamp electrophysiology, RNAscope in situ hybridization, immunostaining, western blot, RT‐PCR, taurine efflux assay, live cell imaging, and stroke model; J.Y. performed acute brain slice patch‐clamp electrophysiology; J.A. and R.R. performed and analyzed LC‐MS experiments; J.C., J.Y., J.C., and K.H.C. analyzed data; J.C., J.Y. and Z.Q. designed the study; and J.C and Z.Q. wrote the paper with input from all authors.

## Supporting information

Supporting Information

## Data Availability

The data that support the findings of this study are available from the corresponding author upon reasonable request.

## References

[1] V. L. Feigin , G. A. Roth , M. Naghavi , P. Parmar , R. Krishnamurthi , S. Chugh , G. A. Mensah , B. Norrving , I. Shiue , M. Ng , K. Estep , K. Cercy , C. J. L Murray , M. H. Forouzanfar , Lancet Neurol. 2016, 15, 913. 27291521 10.1016/S1474-4422(16)30073-4

[2] M. A. Moskowitz , E. H. Lo , C. Iadecola , Neuron 2010, 67, 181. 20670828 10.1016/j.neuron.2010.07.002 PMC2957363

[3] P. M. George , G. K. Steinberg , Neuron 2015, 87, 297. 26182415 10.1016/j.neuron.2015.05.041 PMC4911814

[4] J. Emberson , K. R. Lees , P. Lyden , L. Blackwell , G. Albers , E. Bluhmki , T. Brott , G. Cohen , S. Davis , G. Donnan , J. Grotta , G. Howard , M. Kaste , M. Koga , R.‐v. Kummer , M. Lansberg , R. I. Lindley , G. Murray , J. M. Olivot , M. Parsons , B. Tilley , D. Toni , K. Toyoda , N. Wahlgren , J. Wardlaw , W. Whiteley , G. J. del Zoppo , C. Baigent , P. Sandercock , W. Hacke , Lancet 2014, 384, 1929. 25106063 10.1016/S0140-6736(14)60584-5 PMC4441266

[5] J. L. Saver , M. Goyal , A. Van der Lugt , B. K. Menon , C. B. Majoie , D. W. Dippel , B. C. Campbell , R. G. Nogueira , A. M. Demchuk , A. Tomasello , P. Cardona , T. G. Devlin , D. F. Frei , R. du Mesnil de Rochemont , O. A. Berkhemer , T. G. Jovin , A. H. Siddiqui , W. H. van Zwam , S. M. Davis , C. Castaño , B. L. Sapkota , P. S. Fransen , C. Molina , R. J. van Oostenbrugge , Á. Chamorro , H. Lingsma , F. L. Silver , G. A. Donnan , JAMA, J. Am. Med. Assoc. 2016, 316, 1279. 10.1001/jama.2016.13647 27673305

[6] B. C. V. Campbell , D. A. De Silva , M. R. Macleod , S. B. Coutts , L. H. Schwamm , S. M. Davis , G. A. Donnan , Nat. Rev. Dis. Primers 2019, 5, 70. 31601801 10.1038/s41572-019-0118-8

[7] M. Fisher , S. I. Savitz , Nat. Rev. Neurol. 2022, 18, 193. 35079135 10.1038/s41582-021-00605-6 PMC8788909

[8] C. Ayata , A. H. Ropper , J. Clin. Neurosci. 2002, 9, 113. 11922696 10.1054/jocn.2001.1031

[9] D. Liang , S. Bhatta , V. Gerzanich , J. M. Simard , Neurosurg Focus 2007, 22, E2. 10.3171/foc.2007.22.5.3 PMC2740913 17613233

[10] R. L. Rungta , H. B. Choi , J. R. Tyson , A. Malik , L. Dissing‐Olesen , P. J. Lin , S. M. Cain , P. R. Cullis , T. P. Snutch , B. A. MacVicar , Cell 2015, 161, 610. 25910210 10.1016/j.cell.2015.03.029

[11] P. A. Sawant‐Pokam , T. J. Vail , C. S. Metcalf , J. L. Maguire , T. O. McKean , N. O. McKean , K. C. Brennan , J. Clin. Invest. 2020, 130, 6005. 33044227 10.1172/JCI134793 PMC7598047

[12] H. Inoue , Y. Okada , J. Neurosci. 2007, 27, 144. 10.1523/JNEUROSCI.4694-06.2007 PMC6673589 17287519

[13] P. J. Feustel , Y. Jin , H. K. Kimelberg , Stroke 2004, 35, 1164. 15017010 10.1161/01.STR.0000124127.57946.a1

[14] H. Inoue , H. Ohtaki , T. Nakamachi , S. Shioda , Y. Okada , J. Neurosci. Res. 2007, 85, 1427. 17394260 10.1002/jnr.21279

[15] Y. Zhang , H. Zhang , P. J. Feustel , H. K. Kimelberg , Exper. Neurol. 2008, 210, 514. 18206872 10.1016/j.expneurol.2007.11.027 PMC2362851

[16] A. Alibrahim , L. Y. Zhao , C. Y. Bae , A. Barszczyk , C. Lf Sun , G. L. Wang , H. S. Sun , Acta Pharmacol. Sin. 2013, 34, 113. 23202801 10.1038/aps.2012.148 PMC4086490

[17] Z. Qiu , A. E Dubin , J. Mathur , B. Tu , K. Reddy , L. J. Miraglia , J. Reinhardt , A. P. Orth , A. Patapoutian , Cell 2014, 157, 447. 24725410 10.1016/j.cell.2014.03.024 PMC4023864

[18] F. K. Voss , F. Ullrich , J. Münch , K. Lazarow , D. Lutter , N. Mah , M. A. Andrade‐Navarro , J. P. Von kries , T. Stauber , T. J. Jentsch , Science 2014, 344, 634. 24790029 10.1126/science.1252826

[19] J. Osei‐Owusu , J. Yang , M. D. C. Vitery , Z. Qiu , Curr. Top. Membr. 2018, 81, 177. 30243432 10.1016/bs.ctm.2018.07.005 PMC6604840

[20] D. Belov Kirdajova , J. Kriska , J. Tureckova , M. Anderova , Front Cell Neurosci. 2020, 14, 51. 32265656 10.3389/fncel.2020.00051 PMC7098326

[21] J. Yang , M. del Carmen Vitery , J. Chen , J. Osei‐Owusu , J. Chu , Z. Qiu , Neuron 2019, 102, 813. 30982627 10.1016/j.neuron.2019.03.029 PMC6685291

[22] M. Balkaya , P. Dohare , S. Chen , A. L. Schober , A. M. Fidaleo , J. W. Nalwalk , R. Sah , A. A. Mongin , Iscience 2023, 26, 106669. 37182109 10.1016/j.isci.2023.106669 PMC10173736

[23] J. J. Zhou , Y. Luo , S.‐R. Chen , J.‐Y. Shao , R. Sah , H.‐L. Pan , Exp Neurol 2020, 332, 113391. 32598930 10.1016/j.expneurol.2020.113391 PMC7398854

[24] C. S. Wilson , P. Dohare , S. Orbeta , J. W. Nalwalk , Y. Huang , R. J. Ferland , R. Sah , A. Scimemi , A. A. Mongin , FASEB J. 2021, 35, e21869. 34469026 10.1096/fj.202002745R PMC8639177

[25] J. M. Simard , M. Chen , K. V. Tarasov , S. Bhatta , S. Ivanova , L. Melnitchenko , N. Tsymbalyuk , G. A. West , V. Gerzanich , Nat. Med. 2006, 12, 433. 16550187 10.1038/nm1390 PMC2740734

[26] N. L. Weilinger , V. Maslieieva , J. Bialecki , S. S. Sridharan , P. L. Tang , R. J. Thompson , Acta Pharmacol. Sin. 2013, 34, 39. 22864302 10.1038/aps.2012.95 PMC4086487

[27] Y. Cai , Y. Zhang , X. Ke , Y. Guo , C. Yao , N. Tang , P. Pang , G. Xie , L. Fang , Z. Zhang , J. Li , Y. Fan , X. He , R. Wen , L. Pei , Y. Lu , Y. Lu , Front Genet 2019, 10, 814. 31681398 10.3389/fgene.2019.00814 PMC6798056

[28] I. H. Hernandez , M. Villa‐Gonzalez , G. Martin , M. Soto , M. J. Perez‐Alvarez , Cells 2021, 10, 1639. 34208834 10.3390/cells10071639 PMC8305833

[29] S. Goebbels , I. Bormuth , U. Bode , O. Hermanson , M. H. Schwab , K. A. Nave , Genesis 2006, 44, 611. 17146780 10.1002/dvg.20256

[30] J. Tao , H. Wu , Q. Lin , W. Wei , X. H. Lu , J. P. Cantle , Y. Ao , R. W. Olsen , X. W. Yang , I. Mody , M. V. Sofroniew , Y. E. Sun , J. Neurosci. 2011, 31, 8306. 21632951 10.1523/JNEUROSCI.0567-11.2011 PMC3500097

[31] J. R. Cook , A. L. Gray , E. Lemarchand , I. Schiessl , J. P. Green , M. C. Newland , D. P. Dyer , D. Brough , C. B. Lawrence , Glia 2022, 70, 1068. 35150591 10.1002/glia.24156 PMC9304177

[32] T. J. Jentsch , Nat. Rev. Mol. Cell Biol. 2016, 17, 293. 27033257 10.1038/nrm.2016.29

[33] T. Fellin , O. Pascual , S. Gobbo , T. Pozzan , P. G. Haydon , G. Carmignoto , Neuron 2004, 43, 729. 15339653 10.1016/j.neuron.2004.08.011

[34] P. Sah , S. Hestrin , R. A. Nicoll , Science 1989, 246, 815. 2573153 10.1126/science.2573153

[35] J. Chu , J. Yang , Y. Zhou , J. Chen , K. H. Chen , C. Zhang , H. Y. Cheng , N. Koylass , J. O. Liu , Y. Guan , Z. Qiu , bioRxiv 2023, 9, eade9931. 10.1126/sciadv.ade9931 PMC10058245 36989353

[36] J. Friard , M. Tauc , M. Cougnon , V. Compan , C. Duranton , I. Rubera , Front Pharmacol 2017, 8, 328. 28620305 10.3389/fphar.2017.00328 PMC5449500

[37] N. H. Bowens , P. Dohare , Y. H. Kuo , A. A. Mongin , Mol. Pharmacol. 2013, 83, 22. 23012257 10.1124/mol.112.080457 PMC3533478

[38] J. Lv , Y. Liang , S. Zhang , Q. Lan , Z. Xu , X. Wu , L. Kang , J. Ren , Y. Cao , T. Wu , K. L. Lin , K. K. Lam Yung , X. Cao , J. Pang , P. Zhou , ACS Chem. Neurosci. 2019, 10, 2786. 30935201 10.1021/acschemneuro.9b00010

[39] W. Deng , R. Mahajan , C. M. Baumgarten , D. E. Logothetis , Pflugers Archiv.‐Eur. J. Physiol. 2016, 468, 817. 26837888 10.1007/s00424-016-1794-9 PMC5317042

[40] Z. C. Ye , N. Oberheim , H. Kettenmann , B. R. Ransom , Glia 2009, 57, 258. 18837047 10.1002/glia.20754 PMC2676787

[41] C. J. Lee , G. Mannaioni , H. Yuan , D. H. Woo , M. B. Gingrich , S. F. Traynelis , J. Physiol. 2007, 581, 1057. 17412766 10.1113/jphysiol.2007.130377 PMC2170820

[42] S. Norn , H. Permin , E. Kruse , P. R. Kruse , Dan Medicinhist Arbog 2014, 42, 99. 25639072

[43] R. H. C. P. Zetterstrom , Acta. Paediatr. 2006, 95, 642. 16754542 10.1080/08035250600719739

[44] B. Chen , W. Lin , W. Qi , S. Li , Z. Hong , H. Zhao , Neuroscience 2020, 444, 64. 32697981 10.1016/j.neuroscience.2020.07.019

[45] F. Liu , D. P. Schafer , L. D. McCullough , J Neurosci Methods 2009, 179, 1. 19167427 10.1016/j.jneumeth.2008.12.028 PMC2674851

[46] A. Chamorro , U. Dirnagl , X. Urra , A. M. Planas , Lancet Neurol. 2016, 15, 869. 27180033 10.1016/S1474-4422(16)00114-9

[47] K. K. Ogden , S. F. Traynelis , Trends Pharmacol. Sci. 2011, 32, 726. 21996280 10.1016/j.tips.2011.08.003 PMC3223280

[48] H. Bading , J. Exp. Med. 2017, 214, 569. 28209726 10.1084/jem.20161673 PMC5339681

[49] T. W. Lai , S. Zhang , Y. T. Wang , Prog. Neurobiol. 2014, 115, 157. 24361499 10.1016/j.pneurobio.2013.11.006

[50] N. B. Hamilton , D. Attwell , Nat. Rev. Neurosci. 2010, 11, 227. 20300101 10.1038/nrn2803

[51] E. B. Malarkey , V. Parpura , Neurochem. Int. 2008, 52, 142. 17669556 10.1016/j.neuint.2007.06.005 PMC2267911

[52] S. Duan , C. M. Anderson , E. C. Keung , Y. Chen , Y. Chen , R. A. Swanson , J. Neurosci. 2003, 23, 1320. 12598620 10.1523/JNEUROSCI.23-04-01320.2003 PMC6742264

[53] Z. C. Ye , M. S. Wyeth , S. Baltan‐Tekkok , B. R. Ransom , J. Neurosci. 2003, 23, 3588. 12736329 10.1523/JNEUROSCI.23-09-03588.2003 PMC6742182

[54] O. Warr , M. Takahashi , D. Attwell , J Physiol 1999, 514, 783. 9882750 10.1111/j.1469-7793.1999.783ad.x PMC2269108

[55] D. O. Kleindorfer , A. Towfighi , S. Chaturvedi , K. M. Cockroft , J. Gutierrez , D. Lombardi‐Hill , H. Kamel , W. N. Kernan , S. J. Kittner , E. C. Leira , O. Lennon , J. F. Meschia , T. N. Nguyen , P. M. Pollak , P. Santangeli , A. Z. Sharrief , S. C. Smith , T. N. Turan , L. S. Williams , Stroke 2021, 52, e364. 34024117 10.1161/STR.0000000000000375

[56] A. Amin , Clin. Interv. Aging 2013, 8, 75. 23378750 10.2147/CIA.S37818 PMC3556861

[57] Nozohouri , A. E. Sifat , B. Vaidya , T. J. Abbruscato , Drug Discov. Today 2020, 25, 535. 31978522 10.1016/j.drudis.2020.01.007 PMC7490972

[58] A. M. Mehta , A. M. Sonabend , J. N. Bruce , Neurotherapeutics 2017, 14, 358. 28299724 10.1007/s13311-017-0520-4 PMC5398992

[59] M. Kramer , J. Dang , F. Baertling , B. Denecke , T. Clarner , C. Kirsch , C. Beyer , M. Kipp , J. Neurosci. Methods 2010, 187, 84. 20064557 10.1016/j.jneumeth.2009.12.020

