# Supplementary material for: The SWELL1 Channel Promotes Ischemic Brain Damage by Mediating Neuronal Swelling and Glutamate Toxicity
Source: Adv Sci (Weinh). 2024 Jul 26;11(36):2401085. doi: 10.1002/advs.202401085 (PMC11423184; doi:10.1002/advs.202401085)
Supplement: Supplementary file 1 — Supporting Information [file ADVS-11-2401085-s001.docx]

**Supplementary Information for**

The SWELL1 channel promotes ischemic brain damage by mediating neuronal swelling and glutamate toxicity

*Jianan Chen^*^, Junhua Yang, Jiachen Chu, Kevin Hong Chen, Jesse Alt, Rana Rais, Zhaozhu Qiu^*^*

*Corresponding author, **Email:** chenjn@zzu.edu.cn (J.C.); zhaozhu@jhmi.edu (Z.Q.)

**This PDF file includes:**

Figures S1 to S7

Tables S1

**
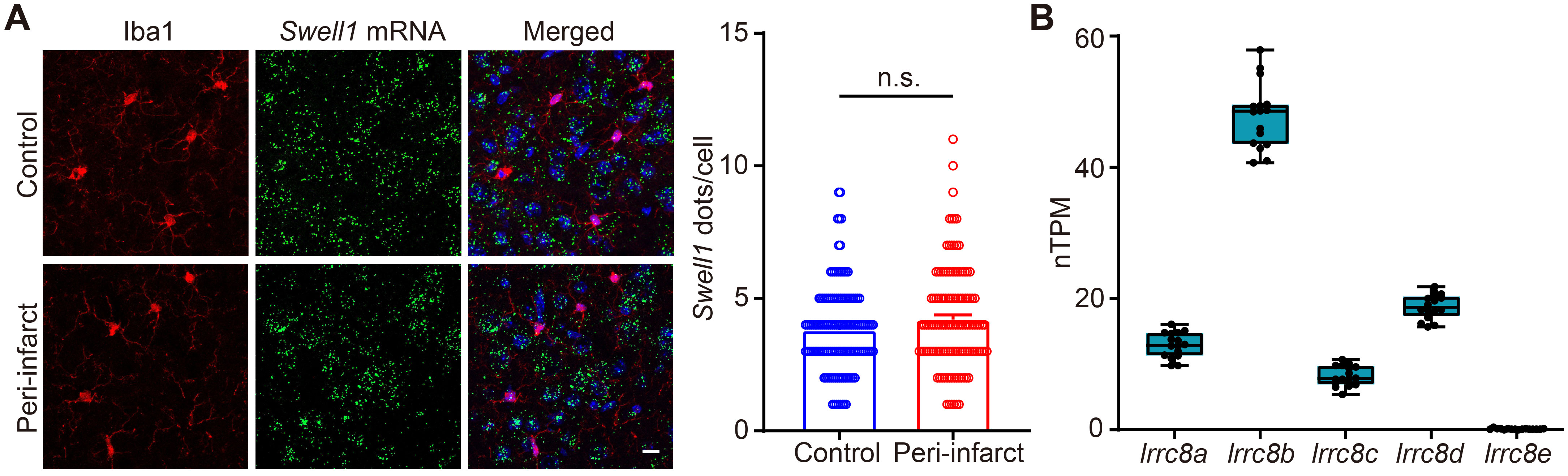
**

**Figure S1. SWELL1 expression in microglia shows no difference after tMCAO.** (**A**) Left: Representative images of *Swell1* RNAscope *in situ* hybridization (green) in control (uninvolved hemispheres) and the peri-infarct regions obtained 8 h after tMCAO. Microglia were immunolabeled with Iba1. Scale bar, 10 μm. Right: Quantification of the SWELL1 expression in Iba1-positive cells. n = 110-118 cells from 3 mice for each group. Data are reported as mean ± SEM. (**B**) Normalized Transcripts per million (nTPM) of LRRC8A-E gene in mouse cerebral cortex tissues from RNA-sequencing data generated by the Human Protein Atlas (www.proteinatlas.org/).

**
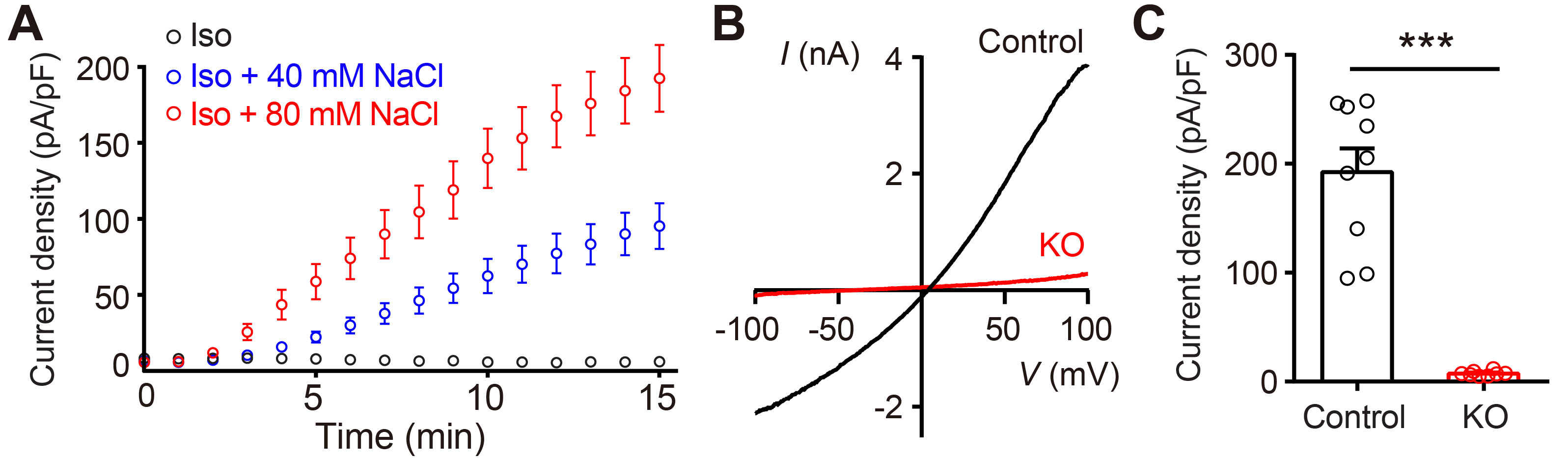
**

**Figure S2. Intracellular hypertonicity activates SWELL1-dependent VRAC currents.** (**A**) Average current densities at +100 mV over time in HeLa cells recorded with a normal internal solution and an addition of 40 mM or 80 mM NaCl. n = 6-9 cells for each group. (**B**) Representative whole-cell currents recorded by ramp protocol from -100 to +100 mV in control and SWELL1 KO HeLa cells with normal internal solution plus 80 mM NaCl. (**C**) Quantification of current densities at +100 mV from (**B**). n = 9 cells for each group. Student’s t tests, ****P* < 0.001.

**
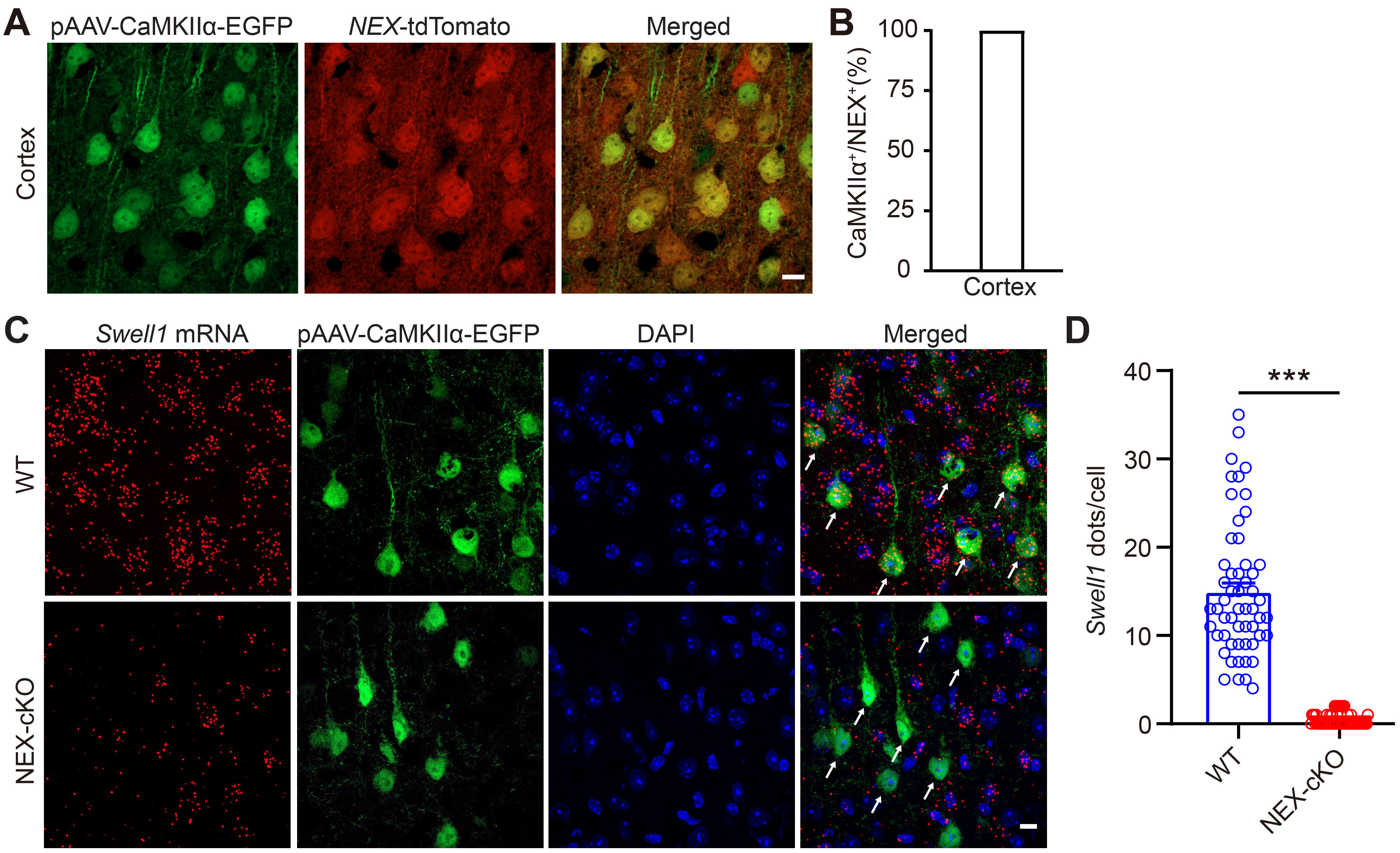
**

**Figure S3. Characterization of NEX-cKO mice.** (**A**) Representative images of brain sections from the cortex of NEX-cre; Ai14^F/+^ mice infected with pAAV-CaMKIIα-EGFP. Scale bar, 10 µm. (**B**) Quantification of the ratio of CaMKIIα^+^/NEX^+^ cells showed complete colocalization between tdTomato and EGFP-positive neurons in the cortex. (**C**) Representative images of *Swell1* RNAscope *in situ* hybridization in the cortex showing the loss of *Swell1* expression in the EGFP-positive neurons from NEX-cKO mice. Arrows indicate EGFP-positive neurons. Scale bar, 10 µm. (**D**) Quantification of *Swell1* mRNA *in situ* hybridization signals in the EGFP positive neurons. n = 55-65 neurons from 3 mice for each group. Mann-Whitney test, ****P* < 0.001. Data are reported as mean ± SEM.


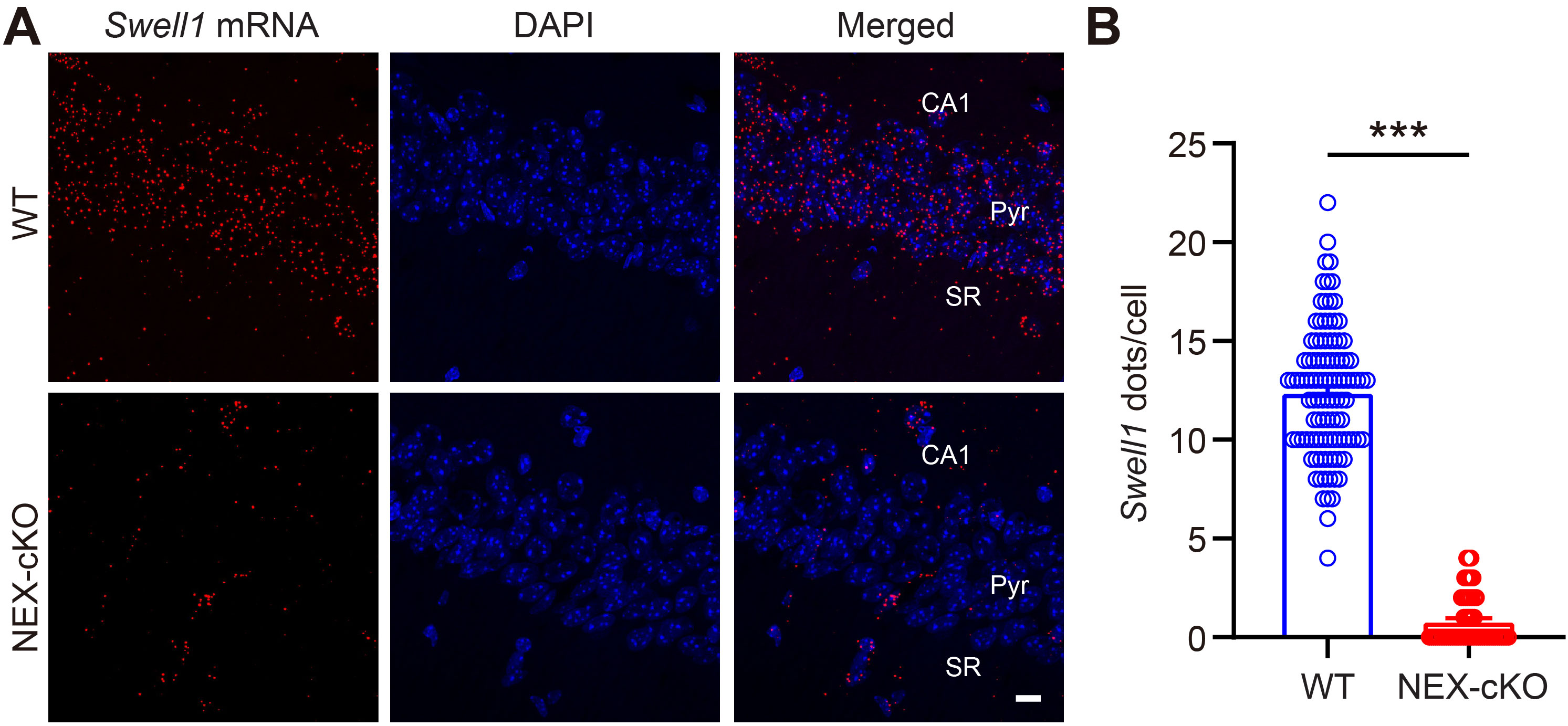
Figure S4. *Swell1* RNAscope *in situ* hybridization in the hippocampus of NEX-cKO mice. (A) Representative images of *Swell1* RNAscope *in situ* hybridization in the hippocampal CA1 region showing loss of SWELL1 expression in the NEX-cKO mice. Pyr, pyramidal cell layer. SR, stratum radiatum. Scale bar, 10 µm. (B) Quantification of *Swell1* mRNA *in situ* hybridization signals in the CA1 cells. n = 60-105 neurons from 3 mice for each group. Mann-Whitney test, ****P* < 0.001. Data are reported as mean ± SEM.

**
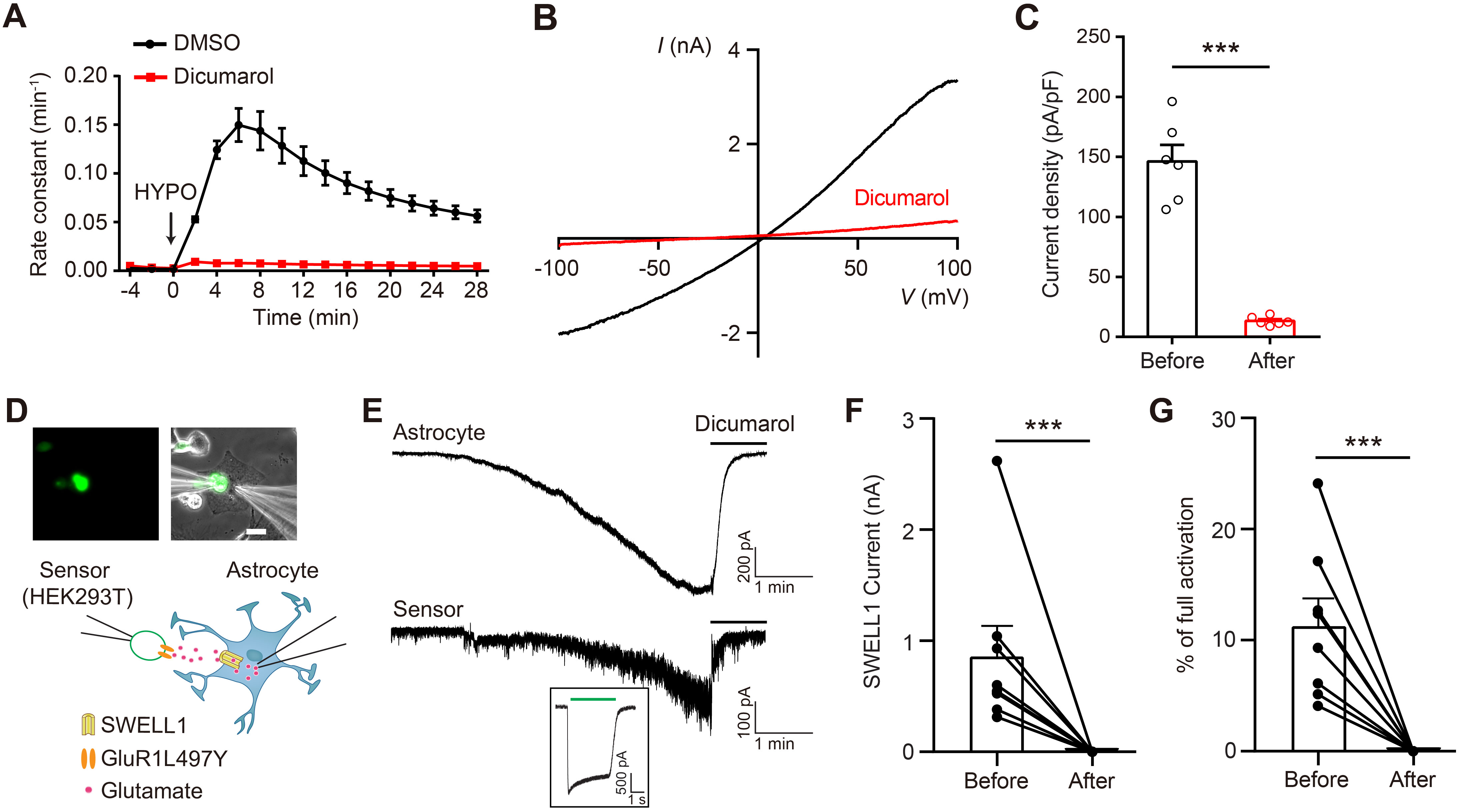
**

**Figure S5. Dicumarol inhibits glutamate release from astrocytes.** (**A**) Time course of the rate constant for swelling-induced [^3^H] taurine efflux in HeLa cells treated with DMSO or Dicumarol (20 μM). Data represent three independent experiments. (**B**) Representative whole-cell currents recorded by ramp protocol from -100 to +100 mV before or after 40 μM Dicumarol treatment in control HeLa cells with normal internal solution plus 80 mM NaCl. (**C**) Quantification of current densities at +100 mV from (**B**). n = 6 cells. Student’s t tests, ****P* < 0.001. (**D**) Representative images (top) and schematic illustration (below) of the sniffer-patch technique. The GFP-positive cell was the HEK293T sensor cell expressing GluR1-L497Y. Astrocyte was whole-cell patched with hypertonic pipette solution (400 mOsm/kg, containing 5 mM glutamate). Scale bar, 20 µm. (**E**) Representative current traces recorded at a holding potential of -60 mV from the WT astrocyte (top) and the sensor cell (below). The full current activation in the sensor cell (inset) was recorded by bath application of 5 mM glutamate (green line). Dicumarol (40 μM) was bath added as indicated. (**F** and **G**) Quantification of SWELL1 currents in the astrocytes (**F**) or the percentage of full activation in the HEK293T sensor cell (**G**) before and after Dicumarol treatment (n = 8 cells). Paired Student’s t tests, ****P* < 0.001. Data are reported as mean ± SEM.


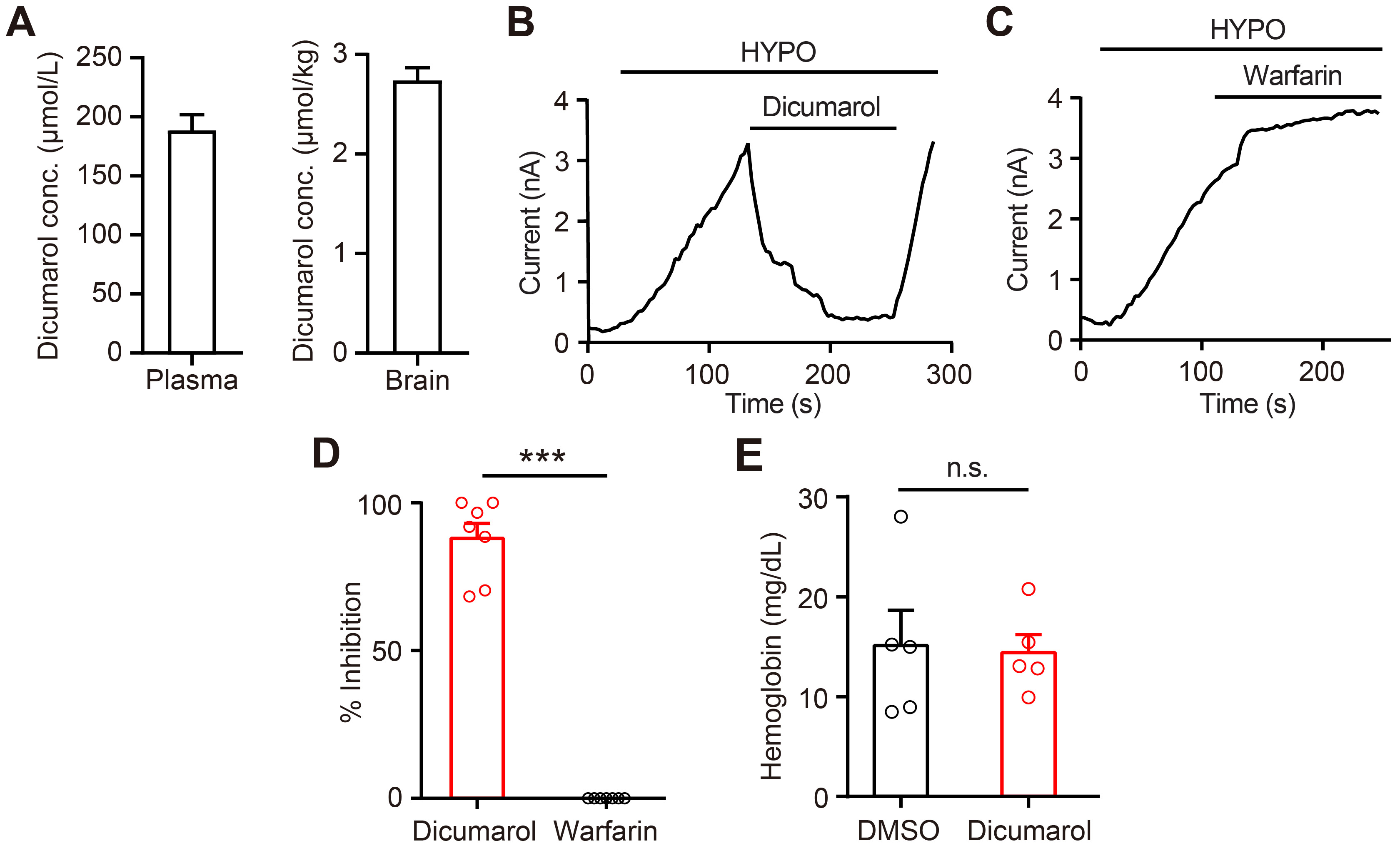
Figure S6. Dicumarol’s mechanism of action on the SWELL1 channel is independent of its inhibition of vitamin K epoxide reductase. (A) Dicumarol concentrations in mouse plasma (left) and brain (right). Blood and brain samples were collected 30 min after intraperitoneal injection of Dicumarol (10 mg/kg). n = 3 mice for each group. (B and C) Time course of whole-cell currents at +100 mV induced by hypotonic solution (HYPO) for primary murine astrocytes. Dicumarol (10 μM) or Warfarin (40 µM) was added as indicated. (D) Quantification of (B) and (C) showed inhibition of SWELL1 currents by Dicumarol, but not Warfarin. n = 7 cells for each group. Mann-Whitney test, ****P* < 0.001. (E) Quantification of the hemoglobin concentration in the stroke hemisphere from Dicumarol- or DMSO-treated mice 1 day after tMCAO. No difference was detected. Data are reported as mean ± SEM.


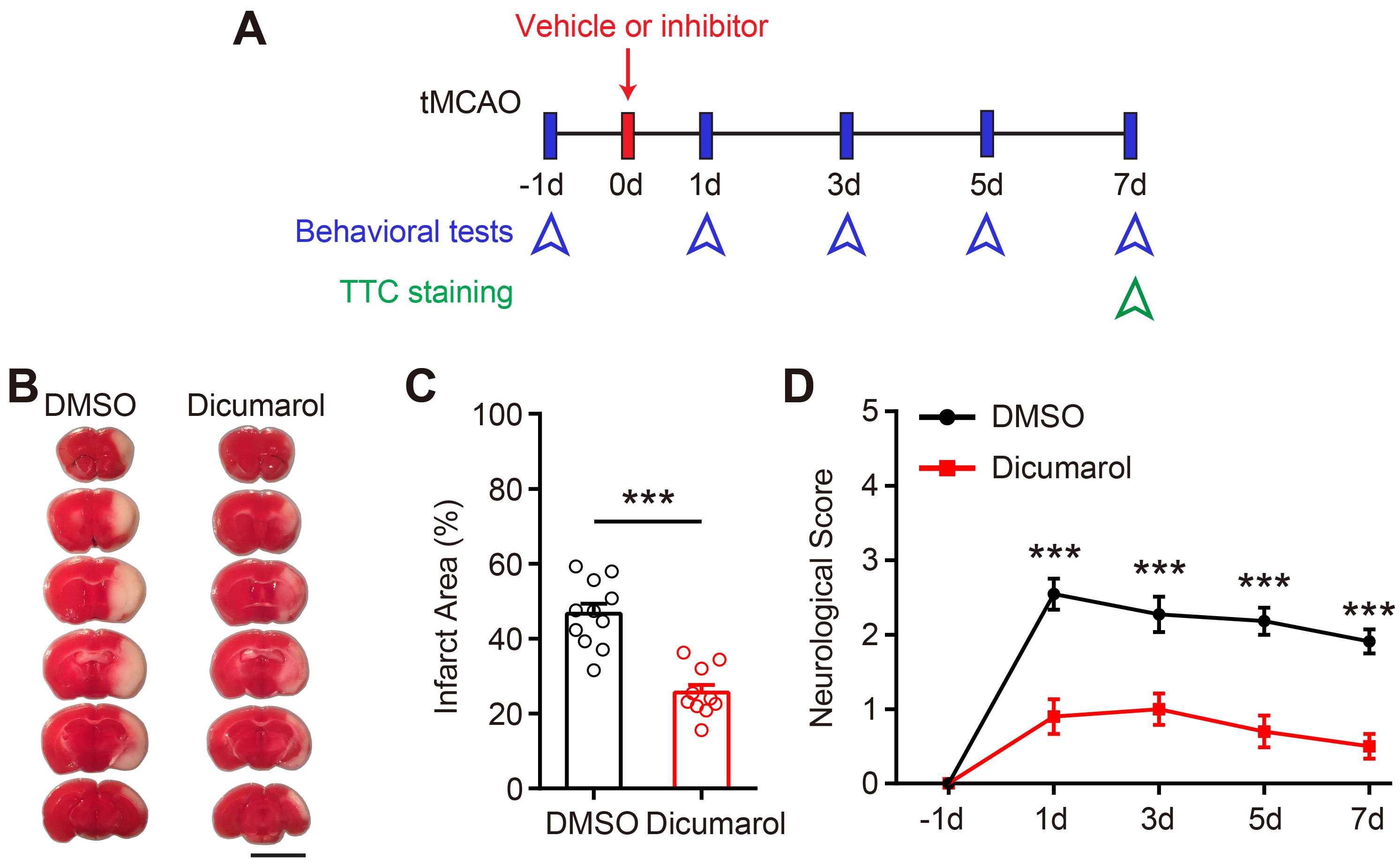
Figure S7. Dicumarol provides persistent neuroprotection over 7 days after ischemic stroke. (A) Experimental design of the ischemic stroke study. 10 μl DMSO (1%) or Dicumarol (100 μM in 1% DMSO) was infused into the brain 20 min before tMCAO in male mice. (B) Representative images of TTC staining 7 days after tMCAO. Scale bar, 5 mm. (C) Quantification of total infarct area volume at day 7 post-tMCAO. n = 10-11 mice for each group. Student’s t test, ****P* < 0.001. (D) Quantification of neurological deficit scores 7 days after tMCAO. n = 10-11 mice for each group. Two-way ANOVA, nonparametric test, ****P* < 0.001. Data are reported as mean ± SEM.

**Table S1. Antibodies and chemicals used in this study**

| **REAGENT** | **SOURCE** | **IDENTIFIER** |
| --- | --- | --- |
| **Antibodies** |  |  |
| Rabbit anti-lba1 | FUJIFILM | Cat# 013-26471 |
| Rabbit anti-Swell1 | This paper | N/A |
| Mouse anti-Beta Actin | Proteintech | Cat# 66009-1-lg |
| TSA Cyanine 3 | AKOYA Biosciences | Cat# NEL744 |
| TSA Cyanine 5 | AKOYA Biosciences | Cat# NEL745 |
| RNAscope Probe-Mm-Lrrc8a | Advanced Cell Diagnostics | Cat# 458371 |
| RNAscope Multiplex Fluorescent Reagent Kit v.2 | Advanced Cell Diagnostics | Cat# 323100 |
| **Chemicals** |  |  |
| TTX | Tocris | Cat# 1069 |
| DL-APV | Sigma-Aldrich | Cat# A8054 |
| DNQX | Sigma-Aldrich | Cat# D0540 |
| Picrotoxin | Sigma-Aldrich | Cat# P1675 |
| Cd^2+^ | Sigma-Aldrich | Cat# 529575 |
| Veratridine | Tocris | Cat# 2918 |
| Biocytin | Sigma-Aldrich | Cat# B4261 |
| D-serine | Sigma-Aldrich | Cat# S4250 |
| L-glutamic acid | Sigma-Aldrich | Cat# G1251 |
| 2,3,5-Triphenyltetrazolium chloride | Sigma-Aldrich | Cat#T8877 |
| Dicumarol | ACROS Organics^TM^ | Cat# 66-76-2 |
| Warfarin | Agilent | Cat# 81-81-2 |
| Bromadiolone | Sigma-Aldrich | Cat# 46035 |
| Coumatetralyl | Sigma-Aldrich | Cat# 45404 |
| Coumestrol | Sigma-Aldrich | Cat# 27883 |
| Coumachlor | Sigma-Aldrich | Cat# 45402 |
| Tioclomarol | Santa cruz biotechnology | Cat# sc-475124 |
| 3-Acetyl-4-hydroxycoumarin | Santa cruz biotechnology | Cat# sc-288892 |
| Ethyl Biscoumacetate | Santa cruz biotechnology | Cat# sc-394088 |
